# Integrated proteotranscriptomics of breast cancer reveals globally increased protein-mRNA concordance associated with subtypes and survival

**DOI:** 10.1186/s13073-018-0602-x

**Published:** 2018-12-03

**Authors:** Wei Tang, Ming Zhou, Tiffany H. Dorsey, DaRue A. Prieto, Xin W. Wang, Eytan Ruppin, Timothy D. Veenstra, Stefan Ambs

**Affiliations:** 1 Molecular Epidemiology Section, Laboratory of Human Carcinogenesis, Center for Cancer Research (CCR), National Cancer Institute (NCI), National Institutes of Health (NIH), Bldg.37/Room 3050B, Bethesda, MD 20892-4258 USA; 2 Laboratory of Protein Characterization, Cancer Research Technology Program, Leidos Biomedical Research, Inc., Frederick National Laboratory for Cancer Research, Frederick, MD USA; 3 Liver Carcinogenesis Section, Laboratory of Human Carcinogenesis, Center for Cancer Research, National Cancer Institute, Bethesda, MD USA; 4 Cancer Data Science Laboratory, Center for Cancer Research, National Cancer Institute, Bethesda, MD USA

**Keywords:** Breast cancer, Proteomics, Gene expression profiling, Systems analysis, Transcription, Survival, African-American

## Abstract

**Background:**

Transcriptome analysis of breast cancer discovered distinct disease subtypes of clinical significance. However, it remains a challenge to define disease biology solely based on gene expression because tumor biology is often the result of protein function. Here, we measured global proteome and transcriptome expression in human breast tumors and adjacent non-cancerous tissue and performed an integrated proteotranscriptomic analysis.

**Methods:**

We applied a quantitative liquid chromatography/mass spectrometry-based proteome analysis using an untargeted approach and analyzed protein extracts from 65 breast tumors and 53 adjacent non-cancerous tissues. Additional gene expression data from Affymetrix Gene Chip Human Gene ST Arrays were available for 59 tumors and 38 non-cancerous tissues in our study. We then applied an integrated analysis of the proteomic and transcriptomic data to examine relationships between them, disease characteristics, and patient survival. Findings were validated in a second dataset using proteome and transcriptome data from “The Cancer Genome Atlas” and the Clinical Proteomic Tumor Analysis Consortium.

**Results:**

We found that the proteome describes differences between cancerous and non-cancerous tissues that are not revealed by the transcriptome. The proteome, but not the transcriptome, revealed an activation of infection-related signal pathways in basal-like and triple-negative tumors. We also observed that proteins rather than mRNAs are increased in tumors and show that this observation could be related to shortening of the 3′ untranslated region of mRNAs in tumors. The integrated analysis of the two technologies further revealed a global increase in protein-mRNA concordance in tumors. Highly correlated protein-gene pairs were enriched in protein processing and disease metabolic pathways. The increased concordance between transcript and protein levels was additionally associated with aggressive disease, including basal-like/triple-negative tumors, and decreased patient survival. We also uncovered a strong positive association between protein-mRNA concordance and proliferation of tumors. Finally, we observed that protein expression profiles co-segregate with a Myc activation signature and separate breast tumors into two subgroups with different survival outcomes.

**Conclusions:**

Our study provides new insights into the relationship between protein and mRNA expression in breast cancer and shows that an integrated analysis of the proteome and transcriptome has the potential of uncovering novel disease characteristics.

**Electronic supplementary material:**

The online version of this article (10.1186/s13073-018-0602-x) contains supplementary material, which is available to authorized users.

## Background

Gene expression profiling of breast tumors has led to the landmark discovery of disease subtypes and novel biomarkers for therapy response and disease survival [1–4]. However, it remains a challenge to define breast cancer biology solely based on gene expression and without knowledge of related changes in the proteome because proteins are key functional drivers of biology and common targets of anticancer drugs. Recent technological advances in mass spectrometry (MS) have laid the groundwork for large-scale characterization of protein expression in human tissues using either untargeted or targeted approaches for protein quantitation [5–8]. System-wide proteomics of the estrogen receptor (ER)-positive disease revealed some insights into disease development that were not revealed by mRNA-based studies [8]. While untargeted proteomics has advanced our knowledge of breast cancer biology [5, 6, 8–13] and other cancers [14–16], a more systematic investigation of the relationship between the tumor proteome and transcriptome, here termed proteotranscriptomic analysis, has the potential to uncover novel molecular alterations in breast cancer biology. To this end, we hypothesized that proteotranscriptomic integration will reveal novel disease characteristics beyond a single technology and applied an integrated analysis of proteomic and transcriptomic data that we jointly collected from human breast tumors and adjacent non-cancerous tissues from patients with survival follow-up. A major difference between this and previous proteome studies is the inclusion of adjacent non-cancerous tissues, African-American patients, and our ability to assess relationships with patient survival. Our study revealed that the proteome and transcriptome describe a partially different tumor biology and that proteins are more commonly upregulated in tumors than the corresponding transcripts. Moreover, our data describe a pathway-centric increase in the concordance between protein and transcript levels that is associated with more aggressive disease and decreased patient survival. These findings were corroborated using proteome and transcriptome data for 404 breast tumors from “The Cancer Genome Atlas” (TCGA) and 77 breast tumors from the Clinical Proteomic Tumor Analysis Consortium (CPTAC) [13, 17].

## Methods

### Tissue collection

Breast cancer patients were recruited between 1993 and 2003, as described previously [18, 19]. Samples of fresh-frozen tumor and adjacent non-cancerous tissue were prepared by a pathologist immediately after surgery and stored at − 80 °C. Clinical and pathological information was obtained from medical records and pathology reports. Details on patient recruitment, specimen collection, and tumor classification are provided in Additional file 1. The collection of biospecimens and the clinical and pathological information was approved by the University of Maryland (UMD) Institutional Review Board (protocol #0298229). The research was also reviewed and approved by the NIH Office of Human Subjects Research Protections (OHSRP #2248).

### Mass spectrometry-based analysis of the proteome

Frozen human tissue samples were pulverized under liquid nitrogen, and extracts for mRNA and protein isolation were prepared. Extracted proteins were digested with trypsin and analyzed using an untargeted MS analysis approach as described in Additional file 1. For the liquid chromatography (LC)-MS measurements, 17 fractions per sample were prepared which generated about 1900 individual fractions from the 118 tissues that were subjected to the MS analysis. The obtained MS data were searched against the UniProt *Homo sapiens* database downloaded from the European Bioinformatics Institute website (ftp://ftp.ebi.ac.uk/pub/databases/integr8) using the Proteome Discoverer 2.0 software (Thermo Fisher Scientific) interfaced with the SEQUEST HT algorithm and filtered with percolator to yield peptide identifications at the 1% false discovery rate (FDR) cutoff. We employed the Protein Scorer and Protein FDR Validator nodes to apply an additional 5% protein-level FDR. Up to two missed tryptic cleavage sites and oxidation of methionyl residues were allowed during this database search. The data was searched with a precursor ion tolerance of 1.4 Da and a fragment ion tolerance of 0.5 Da and two levels of grouping were applied, one for peptide grouping and one for protein grouping. We selected the “strict maximum parsimony principle” option, and only the best ranked peptide-spectrum match (PSM) per spectrum was used for protein identification and grouping. To further reduce false-positive discovery, we considered only those proteins as correctly identified when at least two peptides in a tissue sample uniquely mapped to these proteins. As the last filtering step that was implemented by us, we calculated protein coverage across all samples (Additional file 2: Figure S1A) and found that the correlation between protein coverage and abundance is very high (rho = 0.97) when we remove those proteins from the analysis that are detected in fewer than 10% (*n* = 12) of the samples (Additional file 2: Figure S1B). By setting this 10% coverage cutoff (after the initial protein level 5% FDR using the Proteome Discoverer 2.0 software), we removed the proteins that are difficult to quantify by our technology, leading to a total of 7141 quantified proteins in 118 tissues that we included into the analyses. This approach was validated by showing that the identified proteins in our study largely overlap with proteins identified in three other studies [8, 12, 13] (Additional file 2: Figure S2). The peptide spectral counts for each tissue are shown in Additional file 3: Table S1. The mass spectrometry proteomics data have been deposited with the ProteomeXchange Consortium (http://proteomecentral.proteomexchange.org) under the dataset identifier PXD005692. To assess differential protein expression between tissues (e.g., tumor vs. non-cancerous tissue), we used the Bioconductor package *DESeq2* that was shown to perform well in label-free MS proteomics [20]. Using *DESeq2*, we estimated the size factor and median values for the ratios of the observed counts, controlled for count differences between samples, and monitored outlier samples using Cook’s distance (Additional file 2: Figure S1C). We then applied negative binomial generalized linear model (GLM) fitting and Wald statistics for significance testing. Furthermore, *DESeq2* implements additional filtering that removes statistically insignificant associations, leading to the preferential removal of proteins with low counts and insignificant differences typically due to high dispersion. *DESeq2* introduces rlog (https://bioconductor.org/packages/release/bioc/vignettes/DESeq2/inst/doc/DESeq2.html#data-transformations-and-visualization), which is calculated by fitting each protein to a GLM with a baseline expression (i.e., intercept only) and computing GLM data for each sample, shrunken with respect to the baseline, using the empirical Bayes procedure. rlog incorporates a prior on the sample differences and removes the dependence of the variance on the mean, particularly the high variance of the count data when the mean is low. After rlog normalization, we found that all samples have a very similar distribution for the transformed proteomic data, as shown in Additional file 2: Figure S1D. To compare the spectral count-based ranking of proteins in our study with the ranking of proteins in the Mertins et al. dataset [13], we plotted *z*-scaled log converted PSMs for each protein common to both studies.

### Gene expression microarray analysis

For gene expression profiling, mRNA was converted into cDNA using the Ambion WT Expression Kit for Affymetrix GeneChip Whole Transcript Expression Arrays (Life Technologies). After fragmentation and labeling using the GeneChip WT Terminal Labeling Kit from Affymetrix, ssDNA was hybridized onto Gene Chip Human Gene 1.0 ST Arrays (representing 28,869 genes) according to Affymetrix standard protocols (Santa Clara, CA). The probe cell intensity data was processed by robust multi-array average (RMA) algorithm and analyzed with the Bioconductor limma R package. For more details, including pathway enrichment analysis, see Additional file 1. We only used protein-coding genes for pathway annotation. The top 20 enriched pathways enriched for upregulated and downregulated protein-coding transcripts are shown in Additional file 4: Table S2.

### Protein-mRNA correlation analysis

A protein-mRNA correlation analysis was performed using the regularized-logarithm transformation (rlog) value of the spectral counts and the normalized log2 probe intensity for mRNAs and is described in detail in Additional file 1. Briefly, we calculated the global Spearman correlation coefficient, rho, for 5677 and 3316 protein-mRNA pairs within tumors and non-cancerous tissues, respectively. Adjusted *P* values based on the analysis of 59 tumors and 38 non-cancerous tissues were computed by the Benjamini-Hochberg procedure [21]. Correlation differences between the tumors and non-cancerous tissues were examined by ranking *rho* for each tissue in the two groups and then performing a Wilcoxon rank sum test. A KEGG (Kyoto Encyclopedia of Genes and Genomes) enrichment analysis was performed using the calculated Spearman correlation coefficients for all protein-mRNA pairs and applying the Kolmogorov-Smirnov test to assess how the concordance between protein/mRNA pairs associates with biological processes. Additional analyses, e.g., relationships with tumor subtypes and mRNA features, are described in Additional file 1.

### Query of The Cancer Genome Atlas breast cancer

Publicly available TCGA/CPTAC breast cancer data were downloaded from the Cancer Genomics Data Server (CGDS, at http://www.cbioportal.org/public-portal). Processing of the data to obtain 70 annotated protein-mRNA pairs for 404 tumors is described in Additional file 1. TCGA/CPTAC proteomics breast cancer data were downloaded together with the corresponding gene expression via cbioportal. The PAM50 assignment for the tumors was obtained from the publicly available data provided by the TCGA analysis group.

### Association between protein expression and shortening of the 3′UTR

We retrieved data from Xia et al. [22], who described 382 genes with significant 3′UTR mRNA shortening in human breast tumors due to alternative polyadenylation based on the analysis of 106 TCGA breast tumor-adjacent tissue pairs.

#### Tumor proliferation score

We selected the array-based gene expression profiles of 11 cell cycle genes (BIRC5, CCNB1, CDC20, CEP55, MKI67, NDC80, NUF2, PTTG1, RRM2, TYMS, UBE2C) and summed them into a metagene score as a marker for tissue proliferation, as described previously [23].

### Non-negative matrix factorization

Non-negative matrix factorization (NMF) was used to describe tumor subgroups with different protein abundance profiles. We selected proteins with the highest variability among the proteins detected in the 59 tumors, using a median absolute deviation cutoff of 0.5, which resulted in 1000 proteins for clustering. We applied the consensus NMF clustering method in the R package (https://cran.r-project.org/web/packages/NMF/index.html) to identify tumor subgroups described by the proteome data. More details describing the tumor proliferation score and the NMF analysis including the survival analysis can be found in Additional file 1.

### Statistical analysis

All statistical tests were two-sided, and an association was considered statistically significant with *P* < 0.05. Statistical analyses were performed using the R software developed by the R Development Core Team at R Foundation for Statistical Computing and packages in Bioconductor [24]. We used paired tests for the statistical analysis of differences in protein and gene expression between tumor-adjacent normal pairs. Survival analysis, e.g., Cox regression and Kaplan-Meier methods, was performed using the *survival* package of R. For correlation analysis, the R function “*cor.test*” was used. We applied the Spearman rank correlation test for protein-mRNA correlations because protein and mRNA abundances do not strictly follow a normal distribution or a linear relationship, consistent with previous observations [25]. Reported Spearman coefficients were corrected for ties. Pearson’s correlation test was applied in the analysis of the relationship between tumor proliferation index and the global protein-mRNA concordance. Lastly, we applied a linear regression model to control for confounders in our correlation analyses of the protein-mRNA concordance with race/ethnicity or disease markers.

## Results

### Proteomic profiling of breast tumors and adjacent non-cancerous tissues

**Fig. 1:**
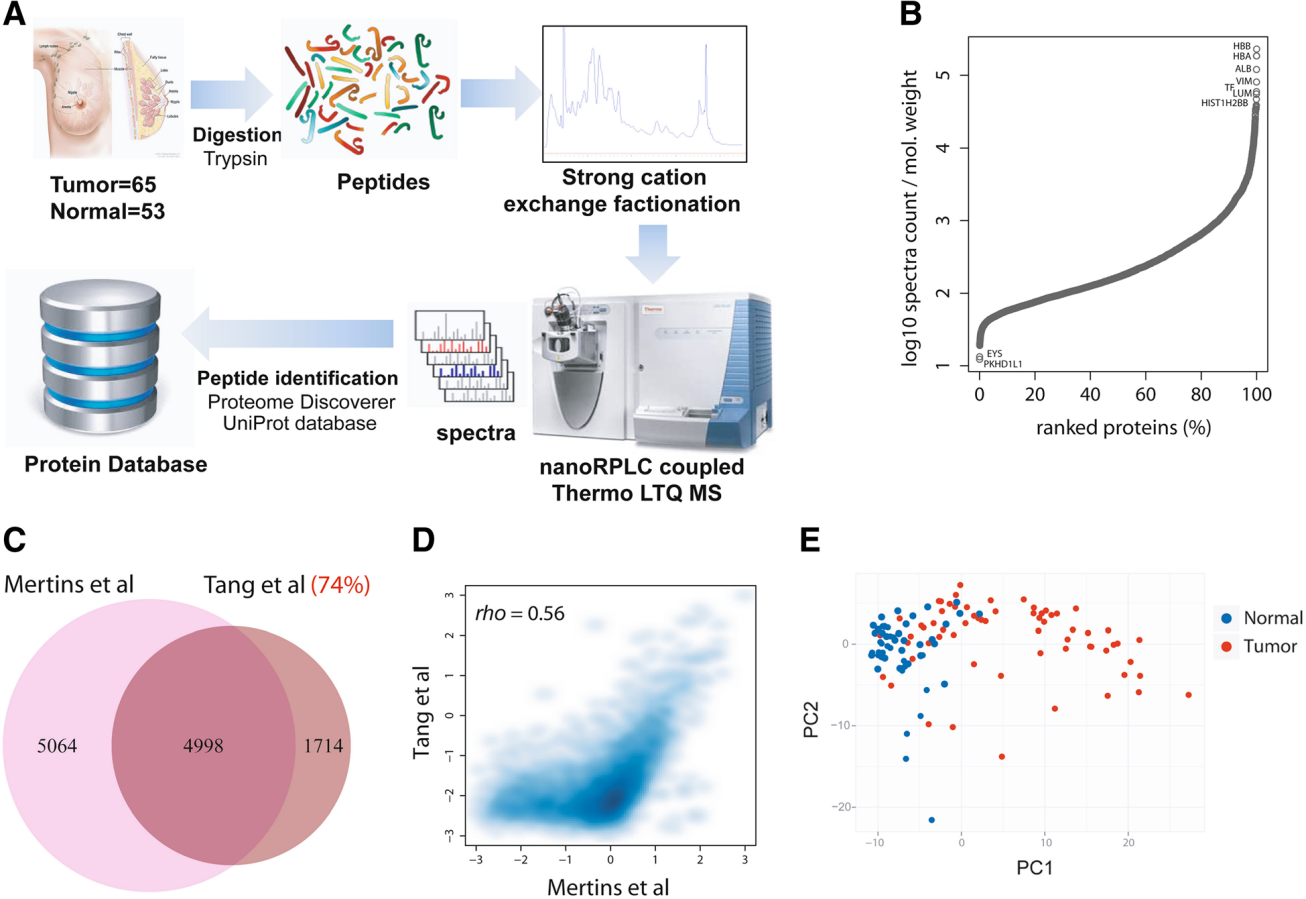
Quantification of proteins in the breast tissues. **a** Mass spectrometry-based proteomics workflow. **b** Dynamic range of protein expression in our study. **c** Overlap in the identified proteins between our study (Tang et al.) and Mertins et al. [13]. **d** Abundance of individual proteins correlates between our study and Mertins et al. (Spearman’s rank correlation rho = 0.56). Correlation is better for high than low abundance proteins. Scaled total spectral counts (*z*-scaled log converted PSMs) were plotted on the *x*- and *y*-axes. **e** A principal component analysis (PCA) based on the abundance of 7141 proteins in 118 tissues (breast tumors *n* = 65; adjacent non-cancerous tissues *n* = 53). The two-dimension PCA plot shows distinct clustering of the tumor (red) and non-cancerous (blue) tissues

**Fig. 2:**
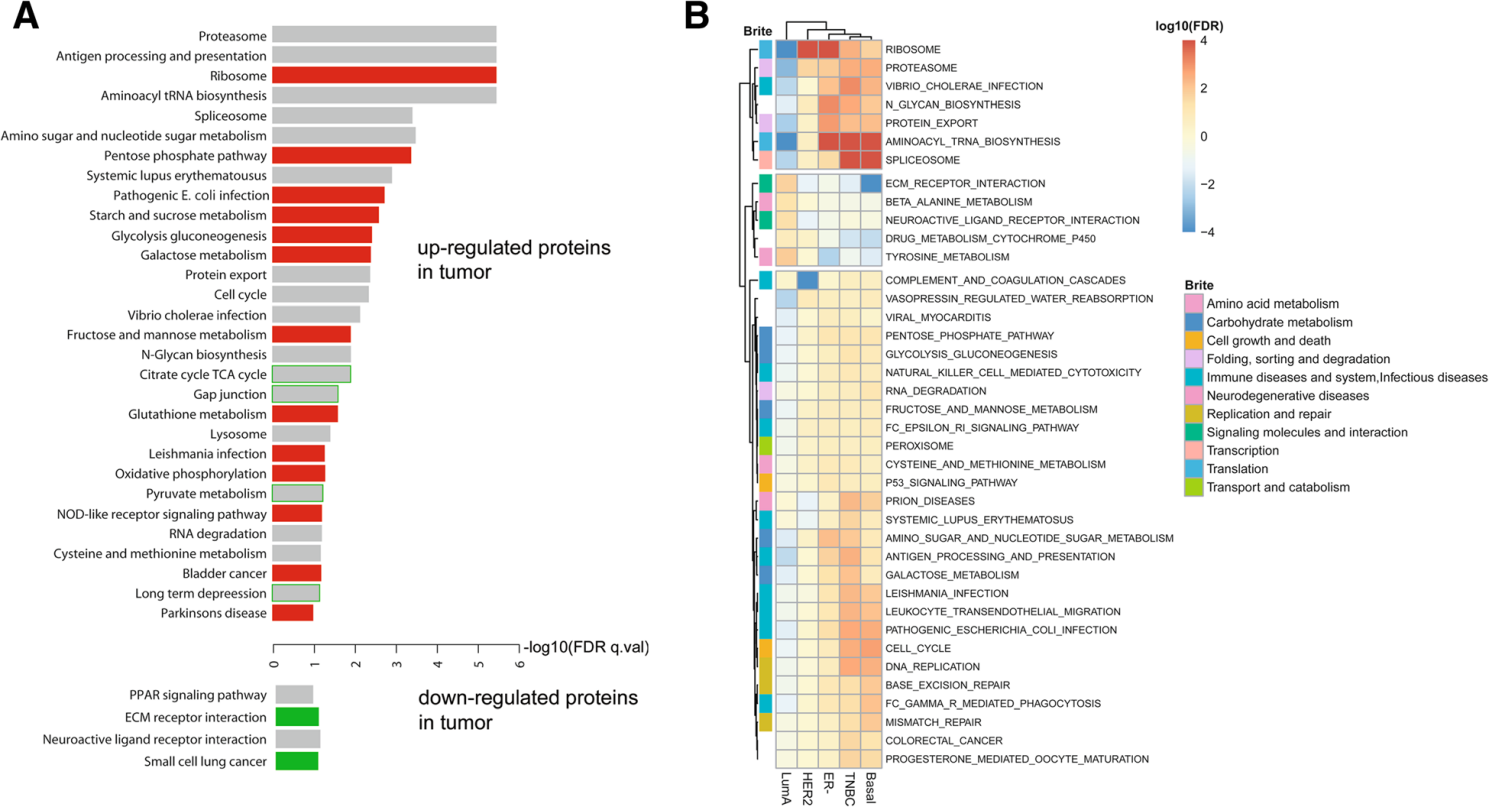
Pathway association of differentially expressed proteins and transcripts. **a** Enrichment of differentially expressed proteins between tumors and adjacent non-cancerous tissue (*n* = 2643; 2165 upregulated and 478 downregulated tumor proteins) in KEGG pathways. Enriched pathways at ≤ 10% FDR are shown for two categories, proteins upregulated (red) and downregulated (green) in tumors. The red and green bars highlight the pathways with enrichment for proteins without a similar enrichment for differentially expressed mRNAs. The gray bars indicate those pathways that were enriched for both proteins and mRNA in tumors. Several metabolism pathways, such as the pentose phosphate pathway, starch and sucrose metabolism, gluconeogenesis and glycolysis, galactose metabolism, and glutathione metabolism, were only enriched for upregulated proteins in tumors but not upregulated mRNAs. Four other pathways, TCA cycle, gap junction, pyruvate metabolism, and long-term depression, showed enrichment for proteins upregulated in tumors whereas pathway-associated mRNAs tended to be downregulated in these same tumors (green-bordered bars). For analysis, all proteins were ranked using Wald statistic and imported into the GSEA pre-ranked module. The KEGG gene sets of MSigDB were selected as the reference database. **b** Heatmap showing subtype-specific enrichment of proteins in KEGG BRITE categories and pathways. Hierarchical clustering using the pathway enrichment scores [FDR-based] yielded three branches with differential enrichment of proteins in KEGG pathways by breast cancer molecular subtype (luminal A, HER2-positive, ER-negative, triple-negative, and basal-like). Shown are unique and common features between subtypes. Red indicates upregulated proteins that are significantly enriched in pathways, whereas blue indicates downregulated proteins with pathway enrichment

We performed a LC-MS-based proteomic analysis that quantified protein abundance, as described under the “Methods” section. The approach generated large-scale proteome data and quantified 7141 proteins in 65 breast tumors and 53 adjacent non-cancerous tissues (Fig. 1a). Patient and tumor characteristics are described in Additional file 5: Table S3, showing the inclusion of both African-American and European-American patients in this study. We stratified tumors into luminal A (estrogen receptor-positive), HER2-positive, and triple-negative/basal-like subtypes, the latter based on both gene and protein marker expression, as described in Additional file 1. The dynamic range of protein expression levels encompassed five orders of magnitude (Fig. 1b). To further validate our coverage of proteins for breast cancer, we compared our list of proteins with the list of identified proteins in three published breast cancer studies [8, 12, 13]. This analysis showed that 70 to 80% of our proteins were shared with each of these studies (Fig. 1c and Additional file 2: Figure S2). This overlap in identified proteins further increased to 86% when we searched for commonly identified proteins between our dataset vs. the combined datasets of Mertins et al. and Tyanova et al. [12, 13]. We also compared the spectral count-based ranking of proteins in our study with the ranking of proteins in the Mertins et al. dataset. This comparison revealed a significant correlation (rho = 0.56), further indicating a high consistency in protein discovery between the two datasets (Fig. 1d). The relative abundance of the 7141 proteins that we quantified in our study separated tumors from the adjacent non-cancerous tissue by principal component analysis (Fig. 1e and Additional file 2: Figure S3) and showed subtype-related protein expression patterns (Additional file 6: Table S4 and Additional file 2: Figure S3). Since both global protein and gene expression data were available for 59 tumors and 38 non-cancerous tissues in our study, we jointly analyzed them for an investigation of the relationship between tumor proteome and transcriptome. This approach showed that differentially expressed proteins between tumor and non-cancerous tissue (*n* = 2643, FDR < 5%) were more frequently upregulated (*n* = 2165, with 1843 proteins at a fold change > 2) than downregulated (*n* = 478, with 270 proteins being downregulated more than twofold) in tumors in a paired analysis of tumor-adjacent normal pairs (Additional file 7: Table S5). In the analysis of gene expression, 58% of the differentially expressed transcripts were upregulated in tumors and 42% showed a decreased expression. The observation that proteins rather than mRNAs are increased in tumors could be related to the shortening of the 3′UTR in cancer cells, which leads to an increased translation of mRNAs into tumor proteins because of the loss of repressive binding sites in these mRNAs [26]. We tested this hypothesis with data from Xia et al., who described 382 genes with significant 3′UTR mRNA shortening in human breast tumors due to alternative polyadenylation [22]. Of the 382 genes, we could map 193 to proteins in our study and found that these proteins have an expression increase in breast tumors (1.77-fold vs. adjacent non-cancerous tissue) more than other proteins (1.41-fold; *P* < 0.05 for difference, Wilcoxon signed-rank test), without an increase in transcript levels (Additional file 2: Figure S4), indicating that 3′UTR shortening leads to increased expression of proteins in breast tumors. Proteins that were significantly upregulated in tumors clustered in distinct biological processes commonly related to protein synthesis and degradation and disease metabolism (Fig. 2a). When we compared the association of upregulated proteins vs. the association of upregulated mRNAs with these processes, only proteins, but not mRNAs, captured ribosome synthesis and function as a disease-associated process. Moreover, only upregulated proteins showed a consistent relationship with metabolic processes in cancer, whereas both upregulated proteins and mRNAs were comparably associated with most other processes (Fig. 2a). We made similar observations when we restricted our analysis to either the basal-like or luminal A subtypes of breast cancer [1, 17]. In basal-like tumors, however, upregulated proteins also clustered in several KEGG pathways related to bacterial and viral infections (Additional file 2: Figure S5), suggesting an activation of infection-related signal pathways in this aggressive subtype. Notably, Mertins et al. also found an enrichment of proteins in immune response/inflammation pathways among basal-like tumors [13]. Together, the two studies advocate that host defense pathways are commonly activated in this tumor subtype, which may relate to an infectious agent contribution in disease etiology. To further capture subtype-specific KEGG pathway enrichment, we performed a hierarchical cluster analysis using the significance of the pathway enrichment scores to generate a heatmap that shows enrichment of proteins in KEGG pathways by tumor subtypes (Fig. 2b). The data reveal that HER2-positive tumors have a distinct downregulation of proteins in the complement and coagulation cascade while triple-negative and basal-like tumors share enrichment for upregulated proteins related to tRNA biosynthesis, spliceosome, cell cycle, and immune diseases and infections and for downregulated proteins related to extracellular matrix (ECM) receptor interactions.

### Increased correlation between protein and mRNA abundance is a disease-associated characteristic

**Fig. 3:**
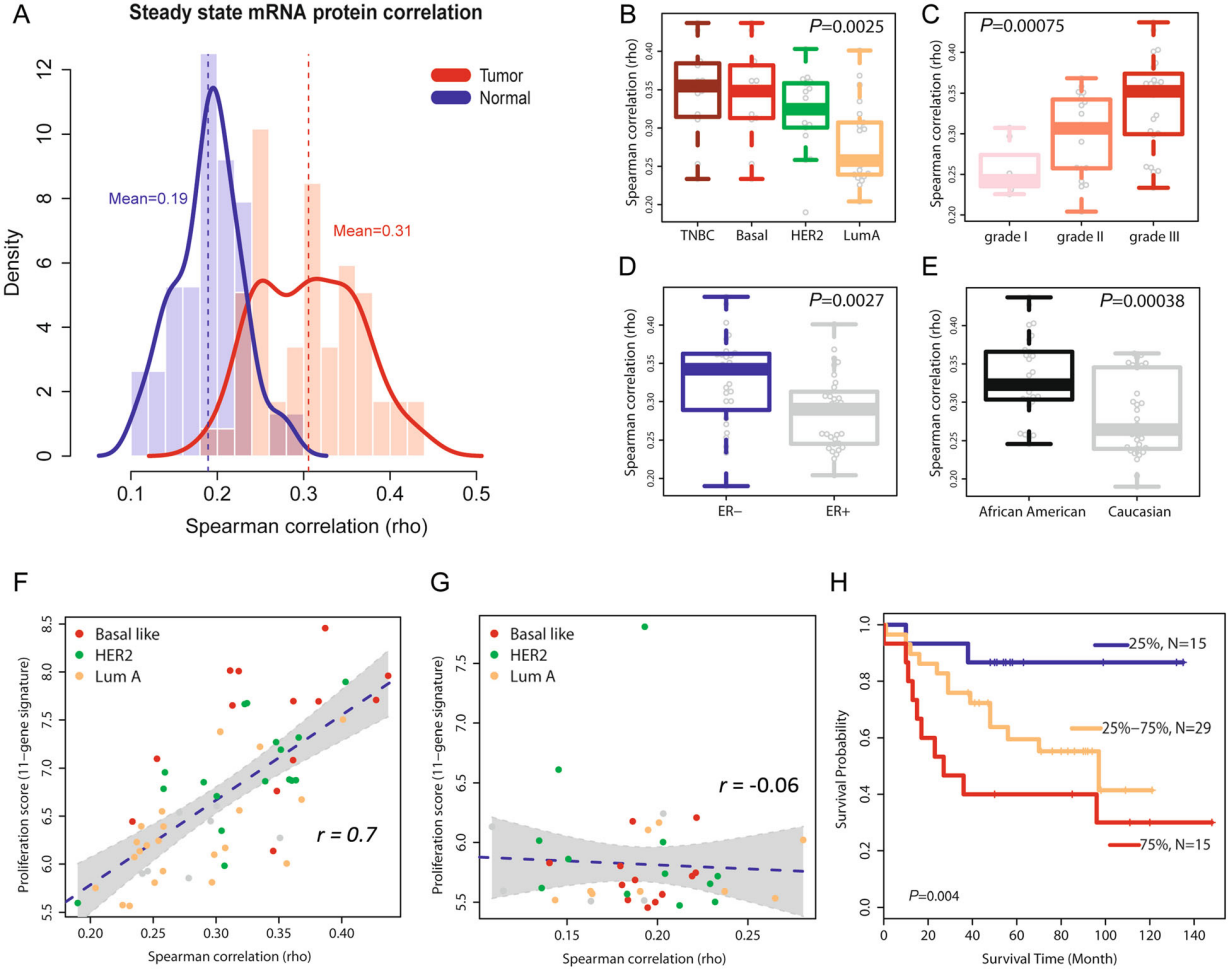
Correlation between steady-state protein and mRNA abundance in breast tumors and association with subtypes and survival. **a** Density plot showing the global Spearman correlation for protein-mRNA pairs within breast tumors (*n* = 59; 5677 protein-mRNA pairs) and adjacent non-cancerous tissue (*n* = 38; 3316 pairs). Protein and mRNA abundance was positively correlated in all tissues samples with a mean Spearman’s correlation coefficient (rho) of 0.31 in tumors, which was significantly higher (*P* = 1.5 × 10^−14^, Wilcoxon rank sum test) than the rho of 0.19 in adjacent non-cancerous tissues. **b** Protein-mRNA pairs have considerably different global correlations among breast cancer subtypes (triple-negative, basal-like, HER2, and luminal A). Triple-negative tumors (TNBC) and tumors of the basal-like subtype (Basal) had the highest mean correlation while luminal A tumors (LumA) had the lowest correlation. **c** Concordance between protein and transcript levels varies by tumor grade. Poorly differentiated grade III tumors had the highest mean correlation between protein and mRNA pairs. **b**, **c** *P* values were calculated using the Kruskal Wallis test. **d**, **e** Estrogen receptor (ER)-negative tumors and tumors from African-American patients show an increased concordance between protein and mRNA abundance. **f** Global protein-mRNA concordance values closely correlate with the proliferation score in the 59 tumors (*r* = 0.7; *P* = 8 × 10^−10^, Pearson correlation) but not in the 38 adjacent non-cancerous tissues (**g**). The dashed line shows regression. **h** High concordance between protein-mRNA pairs in tumors is associated with decreased breast cancer survival. Tumors were stratified into three groups according to their global correlation coefficient across all protein-mRNA pairs [< 25%, 25–75%, and > 75% (tumors with highest global correlation)]. Shown is a Kaplan-Meier plot for these three groups. Tumors with the highest correlations between protein and mRNA pairs had the worst outcome [Cox regression hazard ratio (HR) comparing 75% vs. 25% tumor group = 6.91, 95% CI 1.5–31.7 adjusted for disease subtypes; *P*_trend_ across the three groups = 0.004]

Next, we examined the relationship between protein and mRNA abundance and its association with disease characteristics. Within breast tissues, the concordance between protein and transcript levels was globally higher in tumors (rho = 0.31) than in adjacent non-cancerous tissues (rho = 0.19) (Fig. 3a). This significant difference (*P* = 1.5 × 10^−14^, Wilcoxon signed-rank test) was not explained by a general difference in protein levels between tumor and non-cancerous tissue because an analysis after stratification of proteins into abundance categories validated the initial finding and showed that independent of protein abundance levels in these tissues, protein-mRNA pairs have a generally increased correlation in cancerous tissues (Additional file 8: Table S6). When we repeated our calculations of the global protein-mRNA concordance for the tumors after random selection of proteome subsets, we obtained results very similar to the full dataset (Additional file 2: Figure S6A). Moreover, when we computed the concordance values in relationship to protein coverage across samples (Additional file 2: Figure S6B) and for the subset of proteins that was detected in both tumors and the adjacent non-cancerous tissues (Additional file 2: Figure S6C-D), the concordance values for the tumors remained significantly increased throughout the range of protein coverage. These findings further underscore the robustness of our data by showing that the observations are independent of the protein abundance across samples. Lastly, we examined if differences in ECM protein expression between tumor and non-cancerous tissue may have confounded this finding. It was shown that the content of ECM proteins can be higher in non-cancerous tissues [8]. Yet, exclusion of 163 annotated ECM proteins from our proteome data did not significantly alter the global protein-mRNA concordance for tumors or the non-cancerous tissues (Additional file 2: Figure S7). Together, these data indicate that the increased correlation between protein and mRNA abundance levels is a disease-associated characteristic. Thus, we asked if this concordance measure is additionally associated with disease aggressiveness and outcome. As shown in Fig. 3b, the concordance between protein-mRNA pairs was highest in the aggressive triple-negative and basal-like tumors, slightly lower in HER2-positive tumors, and lowest in luminal A tumors. Likewise, the concordance increased with a more undifferentiated disease grade (Fig. 3c), and both ER-negative tumors and tumors from African-American patients had a significantly higher global concordance for protein-mRNA pairs than either ER-positive tumors or tumors from European-American patients, respectively (Fig. 3d and e). To examine the possibility of confounding in these observations, we applied a multivariable regression analysis. This test showed that the difference in protein-mRNA correlation between African-American and European-American patients is independent of tumor subtypes and grade (*P* = 0.005). In addition, we found that the association of the protein-mRNA correlation with disease grade was independent of the tumor ER status (*P* = 0.013) while the relationship of the protein-mRNA correlation with the ER status was partly confounded by disease grade (*P* = 0.053). In our concluding analysis, we identified 285 proteins whose expression level correlated with disease grade (Additional file 2: Figure S8 and Additional file 9: Table S7). These proteins were enriched for highly correlated protein-gene pairs (285 pairs; mean rho = 0.38) and were functionally associated with protein metabolism, spliceosome and ribosome functions, immune response and infections, and extracellular matrix-receptor interactions.

### High concordance between protein-mRNA pairs in tumors is associated with decreased breast cancer survival

**Fig. 4:**
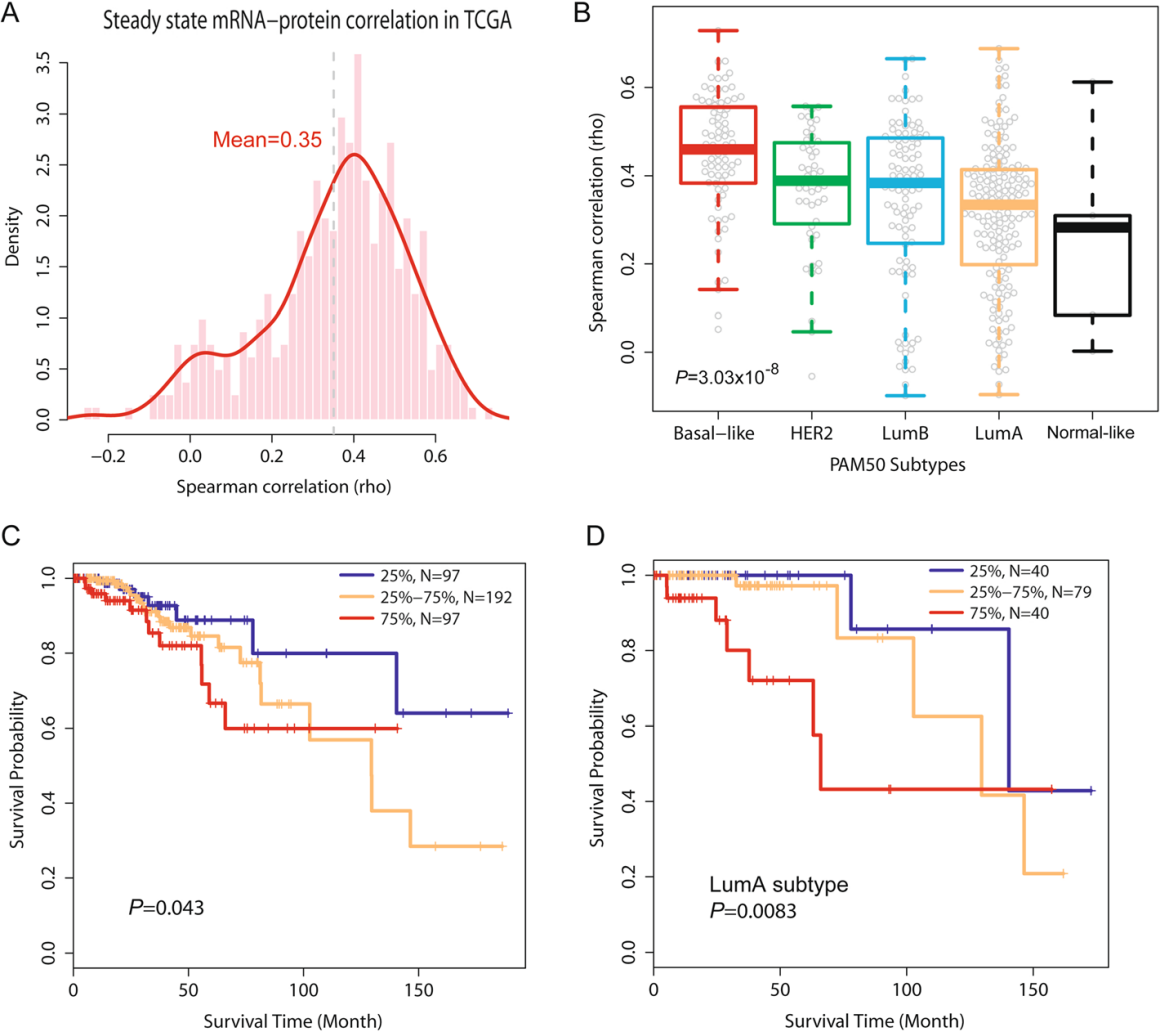
Global concordance between protein-mRNA pairs in TCGA breast tumors and association with molecular subtypes and survival. **a** Density plot showing global Spearman correlation for 70 protein-mRNA pairs within 404 TCGA breast tumors. **b** Significantly different global correlations for protein-mRNA pairs among the molecular breast cancer subtypes (*P* = 3 × 10^−8^, Kruskal Wallis test), with basal-like tumors having the highest mean correlation. Shown are the PAM50-defined subtypes for TCGA tumors. **c** Global concordance between protein-mRNA pairs in TCGA tumors is associated with survival. Stratification of tumors into three groups [< 25%, 25–75%, and > 75% (tumors with highest global correlation)]. Tumors with the highest mean correlation between protein and mRNA pairs had the worst outcome [HR 75% vs. 25% tumor group = 2.6, 95% CI 1.01–6.65; *P*_trend_ across the three groups = 0.043]. **d** Increased global concordance between protein-mRNA pairs is associated with reduced survival in patients with luminal A tumors. *P*_trend_ = 0.0083

To uncover functional correlates between increased protein-mRNA concordance in tumors and tumor biology, we assessed the proliferation level of each tumor by computing an expression metagene score comprised of 11 cell cycle genes (see the “Methods” section) and correlated this score with a protein-mRNA concordance. The analysis revealed a strong positive association between proliferation scores and protein-mRNA concordance [*r* = 0.7, unadjusted Pearson correlation; *r* = 0.59 after adjusting for disease subtypes (*P* = 2.5 × 10^−7^)] in tumors (Fig. 3f and Additional file 10: Table S8), but this relationship did not exist in the adjacent non-cancerous tissues (Fig. 3g). In a second approach, we applied gene set enrichment analysis (GSEA) to characterize concordant protein-mRNA pairs. GSEA showed that ribosomal proteins and genes in the “cell cycle” KEGG pathway were the most significantly enriched ones among the highly correlated protein-mRNA pairs. Having observed that a globally increased protein-mRNA concordance is a characteristic of disease aggressiveness, we asked if it influences disease survival as well. We grouped patients according to their tumor protein-mRNA concordance scores and compared survival of patients with the lowest mean scores in their tumors (< 25%) to patients with either intermediate (25 to 75%) or the highest mean scores (> 75%) in their tumors (Fig. 3h). The survival analysis revealed that a globally increased concordance between protein-mRNA pairs in tumors is significantly associated with reduced survival (*P*_trend_ = 0.004), and tumors with the highest concordance scores conferred a significantly increased risk of an early cancer death when compared to tumors with the lowest concordance scores [hazard ratio (HR) 6.91, 95% confidence interval (CI) 1.5–31.7; *P* = 0.013] (Fig. 3h). This association of the protein-mRNA concordance with patient survival was independent of the tumor proliferation score (HR 7.59, 95% CI 1.25–46.2; *P* = 0.028), as shown by a Cox regression analysis with the proliferation score as covariable (see also Additional file 11: Table S9). Our observations were validated in the TCGA breast cancer dataset [17] with an analysis of 404 tumors with reverse phase protein array (RPPA) data for 70 informative protein-mRNA pairs and patient survival information (Fig. 4a–d and Additional file 1) and in the CPTAC breast cancer proteomics dataset, consisting of high-quality proteome and corresponding gene expression data for 77 tumor samples but limited outcome data [13] (Additional file 2: Figure S9). The concordance between protein-mRNA pairs was highest in the most aggressive molecular subtypes, basal-like and HER2-enriched, and lowest in the least aggressive molecular subtypes, luminal A and normal-like (Fig. 4b and Additional file 2: Figure S9), in agreement with the findings in our discovery dataset. Moreover, an increased global concordance between protein-mRNA pairs in the breast tumors was again associated with reduced survival (Fig. 4c and Additional file 11: Table S9). When we performed an additional analysis restricted to luminal A tumors, the most common breast cancer subtype, an increased protein-mRNA concordance in these tumors defined disease aggressiveness and was also significantly associated with reduced patient survival (Fig. 4d and Additional file 11: Table S9).

### Characteristics of proteins and mRNAs with increased protein-mRNA correlations in tumors

**Fig. 5:**
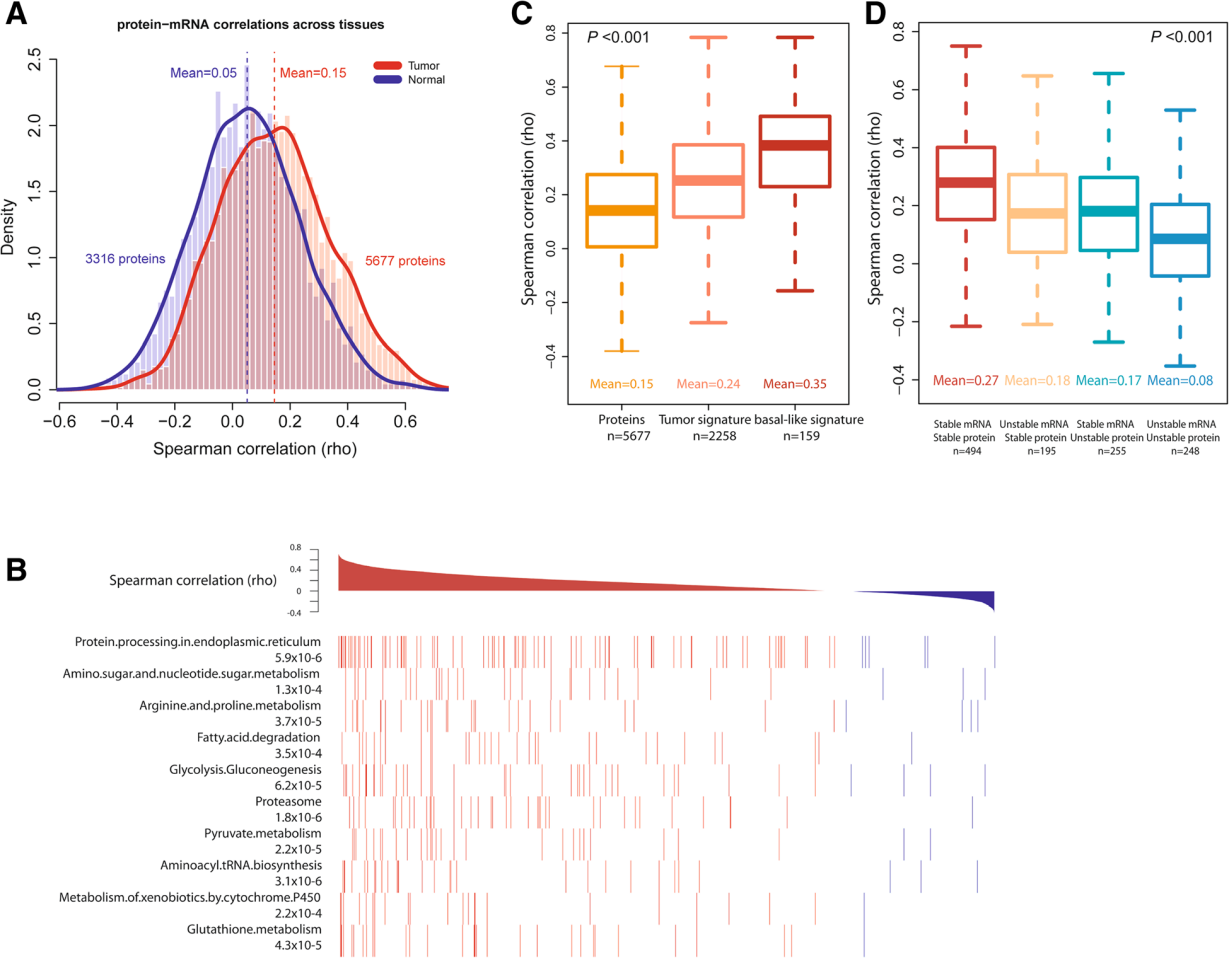
Global protein-mRNA correlations across breast tissues and their association with tumor characteristics. **a** Density plot for global Spearman correlation between protein and mRNA pairs across tumors (*n* = 59; 5677 protein-mRNA pairs) and adjacent non-cancerous tissue (*n* = 38; 3316 pairs). Mean global correlation coefficients are significantly different between tumor and adjacent non-cancerous tissue (*P* < 2.2 × 10^−16^, Wilcoxon rank sum test). **b** Concordance between protein and mRNA pairs is associated with discrete pathways in KEGG, e.g., protein processing and metabolism-related pathways. Protein-mRNA pairs for these pathways tend to show significantly increased concordances. Shown are the 10 highest ranked KEGG pathways that are enriched for high concordance protein-mRNA pairs. Multiple test-adjusted *P* values from the Kolmogorov-Smirnov test. **c** Protein-mRNA correlation for all proteins in tumors (“proteins,” 5677 protein-mRNA pairs), for tumor proteins with significant differences in abundance between tumor and adjacent non-cancerous tissue (“tumor signature,” *n* = 2258 pairs), and for basal-like tumor proteins with significant differences in abundance between tumor and adjacent non-cancerous tissue (“basal-like signature,” 159 pairs). Basal-like signature protein-mRNA pairs have the highest concordance. Definition of the signatures is described in the “Methods” section. **d** Concordance between protein and mRNA levels in breast tumors is associated with the predicted stability of proteins and mRNAs. Protein-mRNA pairs consisting of a protein and mRNA that are both stable have the highest mean concordance

To obtain additional insight into features that may affect protein-mRNA concordance, we applied an across-subject correlation matrix (described in Additional file 1) and calculated protein-mRNA concordance across tumors or the adjacent non-cancerous tissues (Additional file 12: Table S10). Correlation levels were weaker in this analysis than in the within tissue analysis, but markedly increased in tumors (Fig. 5a), analog to observations in the TCGA colorectal cancer study [16]. Key observations from this analysis included the striking finding that protein-mRNA pairs with a high positive correlation in tumors clustered prominently in pathways related to protein processing and tumor metabolism (Fig. 5b). Moreover, proteins with a differential abundance between tumor and adjacent non-cancerous tissue (“tumor signature,” Additional file 12: Table S10) had a higher mean protein-mRNA coefficient than the pool of all detected proteins (Fig. 5c), whereas those proteins that were significantly differently expressed between basal-like tumors and adjacent non-cancerous tissue (“basal-like signature,” Additional file 12: Table S10) showed the highest correlation. To find characteristics of proteins and mRNAs that increase protein-mRNA correlations in tumors, we grouped protein-mRNA pairs by predicted stability for both (described in Additional file 1) and found that the global correlation coefficient between protein-mRNA pairs increased with the predicted stability of these molecules (Fig. 5d), consistent with a previous observation [16, 27]. In summary, we found that increased protein-mRNA correlations are a disease marker that is pathway-centric and concentrates in metabolism-related pathways and is moderately influenced by predicted mRNA stability.

### Proteomic subtypes and their association with Myc signaling

**Fig. 6:**
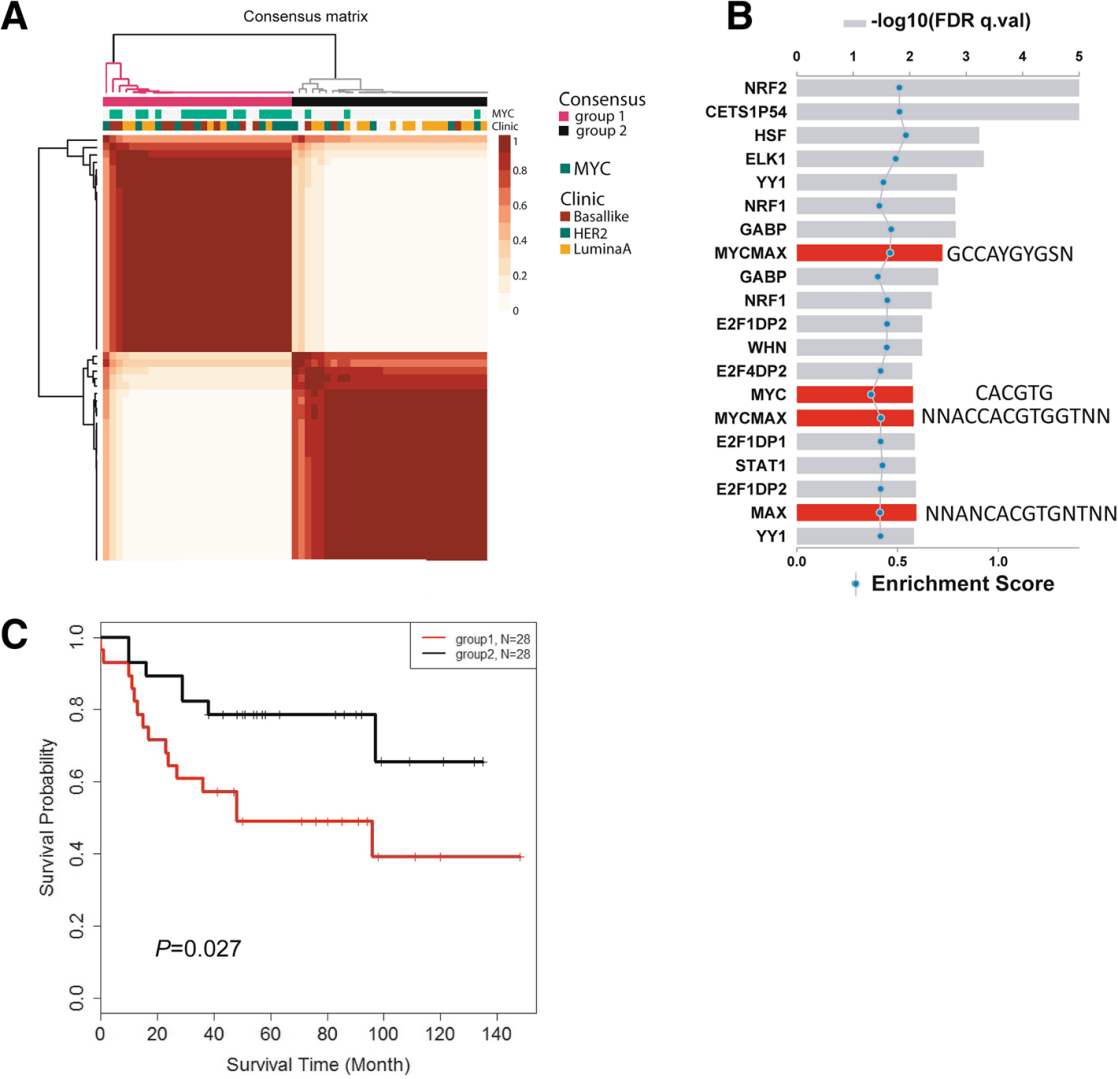
Protein abundance profiles separate breast tumors into two groups with greatly different patient survival. **a** Consensus matrix plot of NMF clustering for 59 breast tumors based on protein abundance levels in these tumors. Two groups of tumors emerged. Group 1 represents tumors enriched for the basal-like subtype (*P* < 0.01, Fisher’s exact test) while group 2 represents tumors enriched for the luminal A subtype (*P* = 0.03). Myc signaling was the strongest classifier for these two tumor groups (*P* < 1 × 10^−8^, Fisher’s exact test); most tumors in group 1 contained a Myc activation signature. For analysis, protein counts were normalized and log transformed, and consensus matrices were computed at *K* = 2–7. NMF class assignment for *K* = 2 was the most robust. The consensus index for each pair of samples is represented by a color gradient from white (0%) to red (100%) in the consensus matrix. **b** Enrichment pattern for transcription factor binding sites in genes that encode differently expressed proteins between tumor groups 1 and 2. Group 1—upregulated proteins are commonly encoded by genes with a predicted Myc binding motif, highlighted in red bars. Different bars represent different Myc binding motifs. GSEA enrichment score is captured by the blue dots. **c** Kaplan-Meier survival analysis comparing tumors in group 1 with tumors in group 2. Survival of patients in group 1 was significantly shorter than the survival of patients in group 2 (log-rank test, *P* = 0.027; HR = 2.65, 95% CI 1.08–6.51, using Cox regression)

Lastly, we examined whether protein abundance profiles can separate breast tumors into distinct subgroups. We applied the NMF algorithm and selected the 1000 proteins with the highest expression variability for clustering (see the “Methods” section). In the best-fit NMF model, two distinct groups of tumors emerged (Fig. 6a and Additional file 2: Figure S10). Group 1 was enriched for basal-like tumors and group 2 for the luminal A subtype. Myc signaling was the strongest differentiator among these two tumor groups, as most tumors in group 1 contained a previously described Myc activation signature [19, 28]. This finding was further supported by the observation that upregulated proteins in group 1 were commonly encoded by genes with a predicted Myc binding motif (Fig. 6b). Our finding indicates a major influence of Myc signaling on the proteome in breast cancer, consistent with the function of Myc as a regulator of ribosome biogenesis and enhancer of protein synthesis [29, 30]. Next, we asked if these two groups of tumors exhibit differences in survival outcomes. As shown in Fig. 6c, patients with group 1 tumors experienced significantly shorter survival than patients with group 2 tumors [hazard ratio (HR) = 2.65, 95% confidence interval 1.08–5.51, group 1 vs. group 2]. Group 1 tumors were also associated with an increased proliferation index and tumor grade, but in contrast to the proteome-defined subtypes, neither the proliferation index nor tumor grade was significant predictors of survival in this dataset.

## Discussion

Here, we provide a comprehensive proteotranscriptomic analysis of breast cancer, including the analysis of tumor-adjacent non-cancerous tissue pairs and patients with survival follow-up, and generate a proteome data resource that includes tumors from African-American and European-American patients. Our data show that mRNA abundance incompletely predicts protein abundance in breast tumors and even less so in the adjacent non-cancerous tissue. Furthermore, the tumor proteome described disease pathways and subgroups that were only partially captured by the tumor transcriptome, consistent with the findings in the CPTAC breast cancer study [13]. Notably, however, our work discovered an increased protein-mRNA concordance in breast tumors as a novel disease characteristic and prognostic factor that is associated with molecular subtypes, aggressiveness, and inferior patient survival.

To the best of our knowledge, a relationship between protein and mRNA abundance as a prognostic marker in cancer has not been previously reported. Concordances between protein-mRNA pairs in breast cancer cell lines have been examined, and a mean correlation score of ~ 0.5 for 94 pairs can be estimated from the study by Kennedy et al. [7]. A more recent study using reverse phase protein arrays reported a mean protein-mRNA correlation score of ~ 0.45 for key cancer proteins across several hundred cell lines and 0.35 for 47 breast cancer cell lines [31], which is comparable with the results from other cell-based studies [27, 32]. Thus, in cultured cells, the transcriptome is a moderate predictor of the proteome. TCGA/CPTAC investigators reported a mean protein-mRNA concordance score of 0.39 for breast tumors and 0.47 for colorectal tumor [13, 16]. The lower average concordance in breast tumors in TCGA/CPTAC and our study could be the result of tumor heterogeneity and variations in technology or could be due to the differences in growth rates between breast and colorectal cancer, as our data show that protein-mRNA concordance in breast tumors is linked to proliferation. The proteogenomic characterization of TCGA/CPTAC colorectal and breast tumors found, as we did, that genes encoding metabolic functions tend to show high protein-mRNA correlations [13, 16], indicating enhanced protein-mRNA coupling in cancer metabolism. This finding indicates that cancer cells require a stricter regulation of their metabolism to survive by linking transcription to immediate translation.

Others examined the proteome of breast cancer and characterized disease subtypes [11–13] or engaged in biomarker discovery [5, 10, 33]. In agreement with the findings by Geiger et al. [5], we also noticed that two candidate prognostic markers, IDH2 and CRABP2, are aberrantly upregulated proteins in breast cancer including basal-like tumors (Additional file 7: Table S5). In contrast, few studies evaluated whether the cancer proteome provides signatures for classification into disease subtypes. In colorectal tumors, proteomic signatures described disease subtypes that partly overlapped with the transcriptome-defined subtypes for this disease [16], while Tyanova et al. [12] reported that hierarchical clustering of breast tumors based on protein expression shows high diversity between tumor samples and no clear separation into the previously reported molecular subtypes [1–4]. In their study, the proteome separated tumors into subgroups enriched for certain subtypes. We observed that the proteome separates human breast tumors into two main clusters with different survival outcomes, where one cluster was enriched for basal-like and the other for luminal tumors. Yet, further analyses showed that a Myc activation signature in breast tumors [19, 28] was the strongest classifier for these two tumor groups in our study, indicating a major influence of Myc signaling on the proteome in breast cancer. This observation is consistent with both the known function of the *MYC* oncogene as a regulator of ribosome biogenesis and enhancer of protein synthesis [29, 30] and the proteogenomic characterization of breast tumors by the TCGA/CPTAC Consortium [13]. In the CPTAC study, *K*-means consensus-based clustering with global proteome data yielded a separation of tumors into three groups, termed basal-enriched, luminal-enriched, and stromal-enriched. While our study using NMF clustering did not distinguish stromal-enriched tumors as a third proteomic subtype, both studies associated the basal-enriched proteomic subtype with Myc activation.

Characterization of breast cancer with either proteome or transcriptome data may yield different insights into tumor biology. Proteins that are upregulated in tumors may associate with processes that are very different from those described by the analysis of upregulated mRNAs. These differences may be partly explained by mRNA properties, such as 3′UTR shortening, leading to increased protein expression without upregulation of mRNA expression in tumors, as our data show. We examined the potential differences between a proteome and transcriptome analysis using tumor-adjacent non-cancerous tissue pairs and jointly examined differentially expressed proteins and mRNAs and their pathway association. Recent studies have demonstrated the advantage of pathway-based analysis in assessing tumor biology [34, 35]. Our approach showed that upregulated proteins specifically cluster in processes related to protein synthesis and degradation and disease metabolism. Proteins, but not mRNA, captured ribosome synthesis and function as a disease-associated process and indicated an activation of infection-related signal pathways in basal-like and triple-negative tumors. The latter is of interest because currently, an infection-related process has not been linked to this subtype. Lastly, HER2-enriched tumors were characterized by a distinct downregulation of proteins in the coagulation cascade, which was not seen on the mRNA level. Thus, the analysis of the proteome can yield insights into tumor biology that are missed by a transcriptome analysis.

## Conclusions

We applied an integrated analysis of proteomic and transcriptomic data that we jointly collected from human breast tumors and adjacent non-cancerous tissues. Our study revealed that the proteome describes differences between cancerous and non-cancerous tissue and disease subtypes that are not captured by the transcriptome. Proteins, but not mRNA, linked infection-related pathways to basal-like and triple-negative breast cancer. We also uncovered cross-omics correlations that we validated in additional datasets. Notably, our work describes an increased protein-mRNA concordance in breast tumors as a disease characteristic that is associated with molecular subtypes, aggressiveness, and inferior patient survival.

## Additional files

Additional file 1: Supplementary materials and methods. (DOC 122 kb)

Additional file 2: **Figure S1.** Processing of proteome data. **Figure S2.** Overlap in identified proteins between our study (Tang et al.) and other proteome datasets for breast cancer. **Figure S3.** Proteome profiles for tumors vs. adjacent non-cancerous tissues and for tumor subtypes. **Figure S4.** Increased expression of proteins in breast tumors encoded by mRNAs with shortened 3′UTR. **Figure S5.** KEGG pathways that are significantly enriched for proteins and mRNAs that were differentially expressed between basal-like tumors and adjacent non-cancerous tissue (13 pairs). **Figure S6.** Robustness of the global protein-mRNA concordance estimates for breast tumors and adjacent non-cancerous tissues. **Figure S7.** Exclusion of extracellular matrix proteins from the proteome dataset does not significantly alter the protein-mRNA concordance calculations for tumors or non-cancerous tissues. **Figure S8**. Correlation between tissue protein levels and tumor grade. **Figure S9.** Correlation between steady-state protein and mRNA abundance (rho) in breast tumors and association with PAM50-defined molecular subtypes using the CPTAC breast cancer proteomics dataset for 77 tumor samples (Mertins et al.). **Figure S10.** Non-negative matrix factorization (NMF) clustering of the tumor proteome data. (PDF 2346 kb)

Additional file 3: **Tables S1.** Peptide spectral counts by best ranked UniProt ID for 118 human breast tissues. (XLSX 7681 kb)

Additional file 4: **Tables S2.** Enrichment of differently expressed protein-coding genes in KEGG pathways (tumor vs. adjacent non-cancerous tissue). (XLSX 13 kb)

Additional file 5: **Tables S3.** Patient characteristics. (DOC 52 kb)

Additional file 6: **Tables S4.** Most differentially expressed proteins between LumA, HER2-positive, and TN/basal-like breast cancer subtypes. (XLSX 195 kb)

Additional file 7: **Tables S5.** Significantly differentially expressed proteins between breast tumors and adjacent non-cancerous tissues (*n* = 52 tissue pairs). (XLSX 636 kb)

Additional file 8: **Tables S6.** Concordance between protein-mRNA pairs in breast tumors and adjacent non-cancerous tissues within six protein abundance categories. (DOC 33 kb)

Additional file 9: **Tables S7.** Proteins (*n* = 285) whose abundance in breast tumors correlated with tumor grade. (XLSX 64 kb)

Additional file 10: **Tables S8.** Proliferation score and global protein-mRNA concordance values for 59 breast tumors. (XLSX 15 kb)

Additional file 11: **Tables S9.** Association of protein-mRNA concordance with survival in the tumor proliferation score-adjusted Cox regression analysis. (DOCX 12 kb)

Additional file 12: **Tables S10.** Concordance values (rho) for 5677 protein-mRNA pairs in breast tumors (*n* = 59). (XLSX 955 kb)

## Abbreviations

CGDS: Cancer Genomics Data Server
CPTAC: Clinical Proteomic Tumor Analysis Consortium
ECM: Extracellular matrix
ER: Estrogen receptor
FDR: False discovery rate
GEO: Gene Expression Omnibus
GLM: Generalized linear model
GSEA: Gene set enrichment analysis
HER2: Human epidermal growth factor receptor 2
HR: Hazard ratio
KEGG: Kyoto Encyclopedia of Genes and Genomes
LC-MS: Liquid chromatography-mass spectrometry
NMF: Non-negative matrix factorization
PCA: Principal component analysis
PSM: Peptide-spectrum match
RPPA: Reverse phase protein array
TCGA: The Cancer Genome Atlas
TNBC: Triple-negative breast cancer
UMD: University of Maryland
UTR: Untranslated region
