# Supplementary material for: Integrated proteotranscriptomics of breast cancer reveals globally increased protein-mRNA concordance associated with subtypes and survival
Source: Genome Med. 2018 Dec 3;10:94. doi: 10.1186/s13073-018-0602-x (PMC6276229; doi:10.1186/s13073-018-0602-x)
Supplement: Supplementary file 1 — Supplementary materials and methods. (DOC 122 kb) [file 13073_2018_602_MOESM1_ESM.doc]

**Supplementary Materials and Methods**

**Tissue collection.** Patients were recruited in Baltimore (Maryland) hospitals between 1993 and 2003, as previously described [1, 2]. Clinical and pathological information (e.g., hormone receptor status) was obtained from medical records and pathology reports. Basal-like and Her2-positive tumors were defined using both gene expression and immunohistochemistry (IHC) data (e.g., ER-negative, HER2-negative, and cytokeratin 5/6-positive or EGFR-positive IHC for basal-like tumors), as previously described for these tumors [2, 3]. Triple-negative tumors were negative for estrogen, progesterone, and HER2 receptor expression, and most were of the basal-like subtype (16 out of 17). Self-reported race/ethnicity was collected as Black (not of Hispanic origin) for African-Americans and White (not of Hispanic origin) for European-Americans. Race/ethnicity was further evaluated with ancestry informative markers and self-identified African-American patients had African ancestry in the range of 67% to 95% [2]. Disease staging was performed according to the tumor–node–metastasis (TNM) system of the American Joint Committee on Cancer/ the Union Internationale Contre le Cancer (AJCC/UICC). The Nottingham system was applied to determine tumor grade. Patient survival was followed up with National Death Index data from the USA [National Center for Health Statistics](https://en.wikipedia.org/wiki/National_Center_for_Health_Statistics).

**Tissue sample preparation for proteome analysis.** Protein extraction and peptide fractionation: Frozen human tissue samples (65 tumors and 53 adjacent non-cancerous tissues) were homogenized in 8 M urea, followed by three rounds of sonication with a 15 second pulse each. After centrifugation of the homogenate at 13,000 rpm for 10 min, the supernatant was loaded on a D-Salt Excellulose desalting column (Pierce Biotechnology, Rockford, IL), eluted with 25 mM NH_4_HCO_3_, pH 8.3, and collected into 0.5 ml fractions. Protein fractions were identified using the Coomassie Plus Assay (Pierce Biotechnology) and were pooled. Total protein concentration of the pooled sample was determined with the BCA protein assay (Pierce Biotechnology). Extracted protein in this sample was digested with trypsin at 37°C overnight using a 1:50 trypsin to protein ratio. Tryptic peptides were desalted using an Empore C18 SPE cartridge (3M). Desalted peptides were fractionated by strong cation chromatography into 45 fractions. Fractions were lyophilized and re-suspended into 0.1% trifluoroacetic acid with a volume that normalized the overall peptide concentration to 0.2 µg/µl. For the liquid chromatography (LC)-mass spectrometry (MS) analysis, fractions 3-43 were combined into 17 fractions per sample, yielding about 1900 individual fractions from the 118 tissues that were subjected to the MS analysis. We performed regular calibration of the MS according to manufacturer’s recommendations and run control samples for quality assurance.

**Mass spectrometry analysis for protein identification**. We used a linear ion trap mass spectrometer (LTQ, Thermo Fisher Scientific, Waltham, MA). LTQ will identify several thousand proteins in a tissue sample [4, 5]. Each of the 1900 fractions (5 µl) was loaded onto an Agilent 1100 nano-capillary HPLC system (Agilent Technologies, Santa Clara, CA) equipped with an in-house made 10 cm integrated nanoRPLC-electrospray ionization emitter column, coupled online with the LTQ. After sample injection, a 20 min wash with 98% mobile phase A (0.1% formic acid) was applied, and peptides were eluted using a linear gradient of 2% mobile phase B (0.1% formic acid in acetonitrile) to 42% mobile phase B within 140 min at a constant flow rate of 250 nl/min. The seven most intense molecular ions in the MS scan were sequentially selected for collision-induced dissociation using normalized collision energy of 35%. The mass spectra were acquired over a mass-to-charge (m/z) range of 300–2000. The nano-source capillary voltage and temperature were maintained at 1.75 kV and 200°C, respectively. Finally, the obtained MS data were searched against the UniProt *Homo sapiens* database downloaded from the European Bioinformatics Institute website (<http://www.ebi.ac.uk/integr8>) using the Proteome Discoverer 2.0 software (Thermo Fisher Scientific) interfaced with the SEQUEST HT algorithm and filtered with percolator to yield peptide lists at a 1% FDR and protein lists at a 5% FDR. In the analysis, up to two missed tryptic cleavage sites and oxidation of methionyl residues were allowed during this database search. The data was searched with a precursor ion tolerance of 1.4 Da and a fragment ion tolerance of 0.5 Da, and two levels of grouping were applied, one for peptide grouping and one for protein grouping. We selected the “Strict Maximum Parsimony Principle” option, only the best rank peptide-spectrum match (PSM) per spectrum was used for protein identification and grouping. As another step to reduce false positive discovery, only those proteins were considered as correctly identified when at least two peptides in a tissue sample uniquely mapped to this protein. As the last filtering step that was implemented by us, we calculated protein coverage across all samples (**Additional file 2:** **Fig. S1A**) and found that the correlation between protein coverage and abundance is very high (⍴ = 0.97) when we remove those proteins from the analysis that are detected in fewer than 10% (n = 12) of the samples (**Additional file 2:** **Fig. S1B**). By setting this 10% coverage cutoff, we removed proteins that are difficult to quantify by our technology, leading to a total of 7141 quantified proteins in 118 tissues that we included into the analyses after using an initial 5% protein-level FDR cutoff. On average, we could quantify 4128 proteins in each tissue, ranging from 2086 to 6199 proteins per tissue, with higher average counts of proteins in tumors. Peptide spectral counts for each tissue are catalogued in **Additional file 3:** **Table S1**. Per setting, only the master protein of this protein group with its UniProt accession was reported by the Proteome Discover software and used as protein ID in **Additional file 3:** **Table S1**. The mass spectrometry proteomics data were deposited with the ProteomeXchange Consortium (http://proteomecentral.proteomexchange.org) in the PRIDE Archive – proteomics data repository under the dataset identifier PXD005692.

**Analysis of the proteome data**. Spectral count is an integer measure and requires tests for analysis that are suited to compare counts. The *DESeq2* package in Bioconductor (<http://bioc.ism.ac.jp/packages/3.1/bioc/html/DESeq2.html>) [6] has been designed for count-based statistical methods and was therefore applied for normalization and comparison. For clustering and principle component analysis (PCA), we transformed the count data using regularized-logarithm transformation (rlog) from the *DESeq2* package (**Additional file 3:** **Table S1** and **Additional file 2:** **Figure S1D**). rlog transforms the original count data into the log2 scale by fitting a model with a term for each sample and a prior distribution for the coefficients which is estimated from the data according to the equation: log2(q_ij_ ) = β_i0_ + β_ij_, in which q_ij_ is a parameter proportional to the expected true concentration of fragments for protein i and sample j, and β_i0_ is an intercept which does not undergo shrinkage, and β_ij_ is the sample-specific effect which is shrunk toward zero based on the dispersion-mean trend over the entire dataset. As q_ij_ represents the part of the mean value divided by the size factor, rlog is better than a variance stabilizing transformation if the size factors vary widely. rlog performs similarly to log2 transformation when proteins have high counts. For proteins with low counts, rlog uses an empirical Bayesian prior on inter-sample differences and behaves approximately homoscedastic. After transformation, we observed very similar distributions across all samples (**Additional file 2:** **Figure S1D**). The rlog value of protein was also used to evaluate protein and mRNA correlation.

To assess differential protein expression between tissues (e.g., tumor vs. non-cancerous tissue), we used a generalized linear model within *DESeq2* that incorporates information from counts and uses negative binomial distribution with fitted mean and a protein-specific dispersion parameter. This analysis has been tested for proteomic data and achieved very high accuracy [7]. It uses estimation of size factors and dispersion, negative binomial GLM fitting, and Wald statistics for significance testing. Benjamini-Hochberg adjustment as implemented in the R was used to correct for multiple testing [8], yielding 2643 proteins that were differentially expressed between the breast tumors and non-cancerous tissues in the study based on a paired analysis of 52 tumor-adjacent normal tissue pairs. For pathway annotation, proteins were ranked by Wald statistic and imported into the GSEA Pre-ranked module. The KEGG gene sets of MSigDB were selected as reference database. Enriched KEGG pathways at a 10% FDR were selected and presented as up-regulated and down-regulated categories. Differently expressed proteins were also ranked by Wald statistic and imported into the GSEA Pre-ranked module for Gene Ontology annotation. Here, the Gene Ontology set of MSigDB was selected as reference database. The Enrichment Map Cytoscape plugin was used to visualize the results for gene-set enrichment in a network based on the GSEA results. We chose the moderately permissive parameter (FDR < 0.1) for gene-set permutation.

**RNA isolation and gene expression microarray analysis.** Total RNA from 59 fresh-frozen tumors and 38 adjacent non-cancerous tissue specimens was isolated using TRIzol reagent according to the manufacturer’s instructions (Life Technologies, Grand Island, NY). RNA integrity for each sample was confirmed with the Agilent 2100 Bioanalyzer (Agilent Technologies, Palo Alto, CA). 250 ng of RNA was converted into cDNA using the Ambion WT Expression Kit for Affymetrix GeneChip Whole Transcript Expression arrays (Life Technologies). After fragmentation and labeling using the GeneChip WT Terminal Labeling Kit from Affymetrix, ssDNA was hybridized onto Gene Chip Human Gene 1.0 ST Arrays (representing 28,869 genes) according to Affymetrix standard protocols (Santa Clara, CA). The probe cell intensity data was processed by RMA algorithm [9], and analyzed with the Bioconductor limma R package (<http://www.bioconductor.org/packages/release/bioc/html/limma.html>). Differentially expressed genes were then assessed using the linear modeling features implemented in limma. For pathway enrichment analysis, genes were ranked by t-statistic and imported into the GSEA Preranked module. The KEGG gene sets of MSigDB were selected as reference. Gene expression data from this study can be found in GEO (http://www.ncbi.nlm.nih.gov/geo) under the accession number GSE39004/GSE37751. Affymetrix Platform: [GPL6244](http://www.ncbi.nlm.nih.gov/geo/query/acc.cgi?acc=GPL6244) [HuGene-1_0-st] Affymetrix Human Gene 1.0 ST Array [transcript (gene) version].

**Protein-mRNA correlation analysis**. We identified 5677 protein-mRNA pairs in the 59 breast tumors (**Additional file 12:** **Table S10**) and 3316 protein-mRNA pairs in the 38 adjacent non-cancerous breast tissues (**Additional file 12:** **Table S10**) that had both protein and mRNA expression data. These proteins were detectable in at least 10% of the tissues and had corresponding mRNA expression. Correlation analyses were performed using either a within-subject or an across-subject correlation matrix. The within-subject approach generates a *rho* for a tumor or adjacent non-cancerous tissue that is a characteristic of a patient that is the most suitable correlate for association studies with clinical features and survival. The across-subject approach generates a *rho* that also captures the variation among subjects and tissue samples and is the more optimal readout studying biological features like pathway enrichment or association with protein/mRNA stability. Within-subject correlations (alternatively called steady state mRNA and protein correlations [10]) were calculated for tumors and non-cancerous tissues, respectively, using the rlog value of protein abundance and normalized log2 probe intensity for mRNAs. We calculated the global Spearman correlation coefficient, *rho*, for the 5677 and 3316 protein-mRNA pairs, within each tumor and non-cancerous tissue, respectively. Adjusted *P* values based on the analysis of 59 tumors and 38 non-cancerous tissues were computed by the Benjamini–Hochberg procedure. Correlation differences between the tumors and non-cancerous tissues were examined by ranking *rho* for each tissue in the two groups and then performing a Wilcoxon rank sum test. The correlation analysis across tissues (alternatively called correlation between mRNA and protein variation [10]) followed the same general analysis approach. Furthermore, we performed a KEGG enrichment analysis using the calculated Spearman correlation coefficients for all protein-mRNA pairs, and applying the Kolmogorov–Smirnov test, to assess how concordance between protein/mRNA pairs associates with biological processes. To calculate mean protein-mRNA correlation for the tumor signature, we first identified 2643 proteins that were differently expressed between 52 tumor-adjacent non-cancerous tissue pairs (FDR < 5%). We then calculated the protein-mRNA correlation for these proteins by gene symbol which yielded 2258 informative protein-mRNA pairs with a mean correlation coefficient of *rho* = 0.24 (tumor signature, **Additional file 12:** **Table S10**). To calculate mean protein-mRNA correlation for a basal-like tumor signature, we first identified 1436 proteins that were differently expressed between 13 basal-like tumor-adjacent non-cancerous tissue pairs at a *P* < 0.05 cutoff (**Additional file 7:** **Table S5**). We then compared protein abundance between 15 basal-like tumors in the study and all other tumors and identified 221 significantly differently expressed genes between them. We then calculated the protein-mRNA correlation for those proteins that were common to both contrasts which yielded 159 informative protein-mRNA pairs with a mean correlation coefficient of *rho* = 0.35 (TN/basal signature, **Additional file 12:** **Table S10**). To investigate the relationship between protein-mRNA correlation and the stability of protein-mRNA pairs, we used a previously described approach [11] and downloaded predicted mRNA and protein half-life data for mouse genes, and defined the top third human ortholog mRNAs and proteins with the highest predicted half-lives as stable mRNAs and proteins, and the bottom third with the lowest predicted half-lives as unstable mRNAs and proteins according to the publication. Thereby, we obtained four categories based on the mRNA and protein half-lives with 494 genes for stable mRNA–stable protein, 195 genes for stable mRNA–unstable protein, 255 genes for unstable mRNA–stable protein, and 248 genes for unstable mRNA–unstable protein.

**Query of The Cancer Genome Atlas (TCGA) breast cancer dataset**. We used R-based APT to access the publicly available TCGA breast cancer data from the Cancer Genomics Data Server (CGDS, at <http://www.cbioportal.org/public-portal>) hosted by the Computational Biology Center at Memorial-Sloan-Kettering Cancer Center. The downloaded data included mRNA expression data (in z-score) and Reverse Phase Protein Array (RPPA) data for 142 proteins including many phosphoproteins, and clinical information from this TCGA dataset. A filtering step to remove low-abundance proteins and phosphoproteins yielded 70 annotated protein-mRNA pairs in 404 tumors for the correlation analysis (TCGA, **Additional file 12:** **Table S10**). We also down-loaded via cbioportal the publicly available TCGA/CPTAC breast cancer proteomics dataset, consisting of high quality proteome and corresponding gene expression data for 77 tumor samples with PAM50 classification [12]. This dataset and the TCGA dataset were then evaluated for protein-mRNA correlations and their association with disease characteristics to obtain validation of findings from the discovery cohort.

**Association between protein expression and shortening of the 3’ UTR.** To examine the relationship between protein expression and 3’ UTR length of the corresponding mRNA in breast cancer, we retrieved data from *Xia et al.* [13], who described 382 genes with significant 3’UTR mRNA shortening in human breast tumors due to alternative polyadenylation based on the analysis of 106 TCGA breast tumor-adjacent tissue pairs. Of the 382 genes, we could map 193 to proteins in our study. We then compared protein expression patterns of these 193 genes with shortened 3’UTR of their mRNAs to the expression of all proteins.

**Tumor proliferation score**. Based on the PAM50 gene panel [14, 15], we selected the array-based gene expression profiles of 11 cell cycle genes (BIRC5, CCNB1, CDC20, CEP55, MKI67, NDC80, NUF2, PTTG1, RRM2, TYMS, UBE2C) and summed them into a meta-gene score as a marker for tissue proliferation, as previously described and termed ROR-P or PAM50 proliferation score [16, 17]. This proliferation signature contains MKI67, the transcript which encodes Ki67, a commonly used proliferation marker with tissue immunohistochemistry.

**Non-negative Matrix Factorization (NMF) to describe tumor subgroups with different protein abundance profiles**. We selected proteins with the highest variability among the 5677 proteins detected in the 59 breast tumors with additional gene expression data (e.g., Myc signaling signature), using a median absolute deviation (MAD) cut-off of 0.5, which resulted in 1000 proteins for clustering. We applied the consensus NMF clustering method in the R package (<https://cran.r-project.org/web/packages/NMF/index.html>) [18] to identify tumor subgroups described by the proteome data. This method computes multiple k-factor factorization decompositions of the protein expression matrix and evaluates the stability of the solutions using a cophenetic coefficient. Consensus matrices and sample correlation matrices were obtained for k=2 to k=6. The final subgroups were defined based on the most stable k-factor decomposition and visual inspection of sample by sample correlation matrices. Clustering with k=2 yielded the most robust clustering results for our data. In the survival analysis, the silhouette width was computed to filter out samples that associated with a subgroup but were not a robust representative of this subgroup, resulting in the removal of 3 samples at a 0.7 cut-off, leading to 56 patients that remained in the survival analysis. Alternatively, we used *ConsensusClusterPlus* (<https://www.bioconductor.org/packages/release/bioc/html/ConsensusClusterPlus.html>) [19] for unsupervised class discovery to identify protein-based tumor subgroups. This approach confirmed the NMF-based findings. Differently expressed proteins between the two groups were also ranked by Wald statistic and imported into the GSEA Pre-ranked module for potential transcription factor annotation. Here, the C3 component of the motif gene set (615 sets) in MSigDB was selected as reference database.

**Statistical analysis**. All statistical tests were two-sided and an association was considered statistically significant with *P* < 0.05. Statistical analyses were performed using the R software developed by R Development Core Team at R Foundation for Statistical Computing and packages in Bioconductor (R version 3.4.1 (2017-06-30)) [20]. Survival analysis, e.g., Cox regression and Kaplan Meier methods, was performed using the *survival* package of R. For correlation analysis, the Spearman rank correlation test was used primarily because protein and mRNA abundances do not strictly follow a normal distribution, in accordance with observations by others [21]. The Pearson’s correlation test was applied in the analysis of the relationship between tumor proliferation index and global protein-mRNA concordance values.

The package features that we used for the study are listed below:

SummarizedExperiment_1.6.3 genefilter_1.58.1 locfit_1.5-9.1 splines_3.4.1 lattice_0.20-35 colorspace_1.3-2 htmltools_0.3.6 stats4_3.4.1 base64enc_0.1-3 blob_1.1.0 survival_2.41-3 XML_3.98-1.9 rlang_0.1.2 DBI_0.7 foreign_0.8-69 BiocParallel_1.10.1 bit64_0.9-7 BiocGenerics_0.22.1 RColorBrewer_1.1-2 matrixStats_0.52.2 GenomeInfoDbData_0.99.0 plyr_1.8.4 stringr_1.2.0 zlibbioc_1.22.0 munsell_0.4.3 gtable_0.2.0 DESeq2_1.16.1 htmlwidgets_0.9 memoise_1.1.0 latticeExtra_0.6-28 Biobase_2.36.2 knitr_1.17 geneplotter_1.54.0 IRanges_2.10.3 GenomeInfoDb_1.12.2 parallel_3.4.1 AnnotationDbi_1.38.2 htmlTable_1.9 Rcpp_0.12.12 acepack_1.4.1 xtable_1.8-2 scales_0.5.0 backports_1.1.0 checkmate_1.8.3 DelayedArray_0.2.7 S4Vectors_0.14.4 Hmisc_4.0-3 annotate_1.54.0 XVector_0.16.0 bit_1.1-12 gridExtra_2.3 ggplot2_2.2.1 pheatmap_1.0.8

**References**

1. Boersma BJ, Howe TM, Goodman JE, Yfantis HG, Lee DH, Chanock SJ, Ambs S: Association of breast cancer outcome with status of p53 and MDM2 SNP309. J Natl Cancer Inst. 2006;98**:**911-9.

2. Terunuma A, Putluri N, Mishra P, Mathe EA, Dorsey TH, Yi M, Wallace TA, Issaq HJ, Zhou M, Killian JK, et al: MYC-driven accumulation of 2-hydroxyglutarate is associated with breast cancer prognosis. J Clin Invest. 2014;124**:**398-412.

3. Nielsen TO, Hsu FD, Jensen K, Cheang M, Karaca G, Hu Z, Hernandez-Boussard T, Livasy C, Cowan D, Dressler L, et al: Immunohistochemical and clinical characterization of the basal-like subtype of invasive breast carcinoma. Clin Cancer Res. 2004;10**:**5367-74.

4. Kline KG, Frewen B, Bristow MR, Maccoss MJ, Wu CC: High quality catalog of proteotypic peptides from human heart. J Proteome Res. 2008;7**:**5055-61.

5. Wisniewski JR, Zougman A, Nagaraj N, Mann M: Universal sample preparation method for proteome analysis. Nat Methods. 2009;6**:**359-62.

6. Love MI, Huber W, Anders S: Moderated estimation of fold change and dispersion for RNA-seq data with DESeq2. Genome Biol. 2014;15**:**550.

7. Langley SR, Mayr M: Comparative analysis of statistical methods used for detecting differential expression in label-free mass spectrometry proteomics. J Proteomics. 2015;129**:**83-92.

8. Benjamini Y, Hochberg Y: Controlling the False Discovery Rate: A Practical and Powerful Approach to Multiple Testing. Journal of the Royal Statistical Society Series B (Methodological). 1995;57**:**289-300.

9. Irizarry RA, Hobbs B, Collin F, Beazer-Barclay YD, Antonellis KJ, Scherf U, Speed TP: Exploration, normalization, and summaries of high density oligonucleotide array probe level data. Biostatistics. 2003;4**:**249-64.

10. Zhang B, Wang J, Wang X, Zhu J, Liu Q, Shi Z, Chambers MC, Zimmerman LJ, Shaddox KF, Kim S, et al: Proteogenomic characterization of human colon and rectal cancer. Nature. 2014;513**:**382-7.

11. Schwanhausser B, Busse D, Li N, Dittmar G, Schuchhardt J, Wolf J, Chen W, Selbach M: Global quantification of mammalian gene expression control. Nature. 2011;473**:**337-42.

12. Mertins P, Mani DR, Ruggles KV, Gillette MA, Clauser KR, Wang P, Wang X, Qiao JW, Cao S, Petralia F, et al: Proteogenomics connects somatic mutations to signalling in breast cancer. Nature. 2016;534**:**55-62.

13. Xia Z, Donehower LA, Cooper TA, Neilson JR, Wheeler DA, Wagner EJ, Li W: Dynamic analyses of alternative polyadenylation from RNA-seq reveal a 3'-UTR landscape across seven tumour types. Nat Commun. 2014;5**:**5274.

14. Parker JS, Mullins M, Cheang MC, Leung S, Voduc D, Vickery T, Davies S, Fauron C, He X, Hu Z, et al: Supervised risk predictor of breast cancer based on intrinsic subtypes. J Clin Oncol. 2009;27**:**1160-7.

15. TCGA Network: Comprehensive molecular portraits of human breast tumours. Nature. 2012;490**:**61-70.

16. Nielsen TO, Parker JS, Leung S, Voduc D, Ebbert M, Vickery T, Davies SR, Snider J, Stijleman IJ, Reed J, et al: A comparison of PAM50 intrinsic subtyping with immunohistochemistry and clinical prognostic factors in tamoxifen-treated estrogen receptor-positive breast cancer. Clin Cancer Res. 2010;16**:**5222-32.

17. Martin M, Prat A, Rodriguez-Lescure A, Caballero R, Ebbert MT, Munarriz B, Ruiz-Borrego M, Bastien RR, Crespo C, Davis C, et al: PAM50 proliferation score as a predictor of weekly paclitaxel benefit in breast cancer. Breast Cancer Res Treat. 2013;138**:**457-66.

18. Gaujoux R, Seoighe C: A flexible R package for nonnegative matrix factorization. BMC Bioinformatics. 2010;11**:**367.

19. Wilkerson MD, Hayes DN: ConsensusClusterPlus: a class discovery tool with confidence assessments and item tracking. Bioinformatics. 2010;26**:**1572-3.

20. Gentleman RC, Carey VJ, Bates DM, Bolstad B, Dettling M, Dudoit S, Ellis B, Gautier L, Ge Y, Gentry J, et al: Bioconductor: open software development for computational biology and bioinformatics. Genome Biol. 2004;5**:**R80.

21. Maier T, Guell M, Serrano L: Correlation of mRNA and protein in complex biological samples. FEBS Lett. 2009;583**:**3966-73.
