# Supplementary material for: Integrated proteotranscriptomics of breast cancer reveals globally increased protein-mRNA concordance associated with subtypes and survival
Source: Genome Med. 2018 Dec 3;10:94. doi: 10.1186/s13073-018-0602-x (PMC6276229; doi:10.1186/s13073-018-0602-x)
Supplement: Supplementary file 2 — Figure S1. Processing of proteome data. Figure S2. Overlap in identified proteins between our study (Tang et al.) and other proteome datasets for breast cancer. Figure S3. Proteome profiles for tumors vs. adjacent non-cancerous tissues and for tumor subtypes. Figure S4. Increased expression of proteins in breast tumors encoded by mRNAs with shortened 3′UTR. Figure S5. KEGG pathways that are significantly enriched for proteins and mRNAs that were differentially expressed between basal-like tumors and adjacent non-cancerous tissue (13 pairs). Figure S6. Robustness of the global protein-mRNA concordance estimates for breast tumors and adjacent non-cancerous tissues. Figure S7. Exclusion of extracellular matrix proteins from the proteome dataset does not significantly alter the protein-mRNA concordance calculations for tumors or non-cancerous tissues. Figure S8. Correlation between tissue protein levels and tumor grade. Figure S9. Correlation between steady-state protein and mRNA abundance (rho) in breast tumors and association with PAM50-defined molecular subtypes using the CPTAC breast cancer proteomics dataset for 77 tumor samples (Mertins et al.). Figure S10. Non-negative matrix factorization (NMF) clustering of the tumor proteome data. (PDF 2346 kb) [file 13073_2018_602_MOESM2_ESM.pdf]

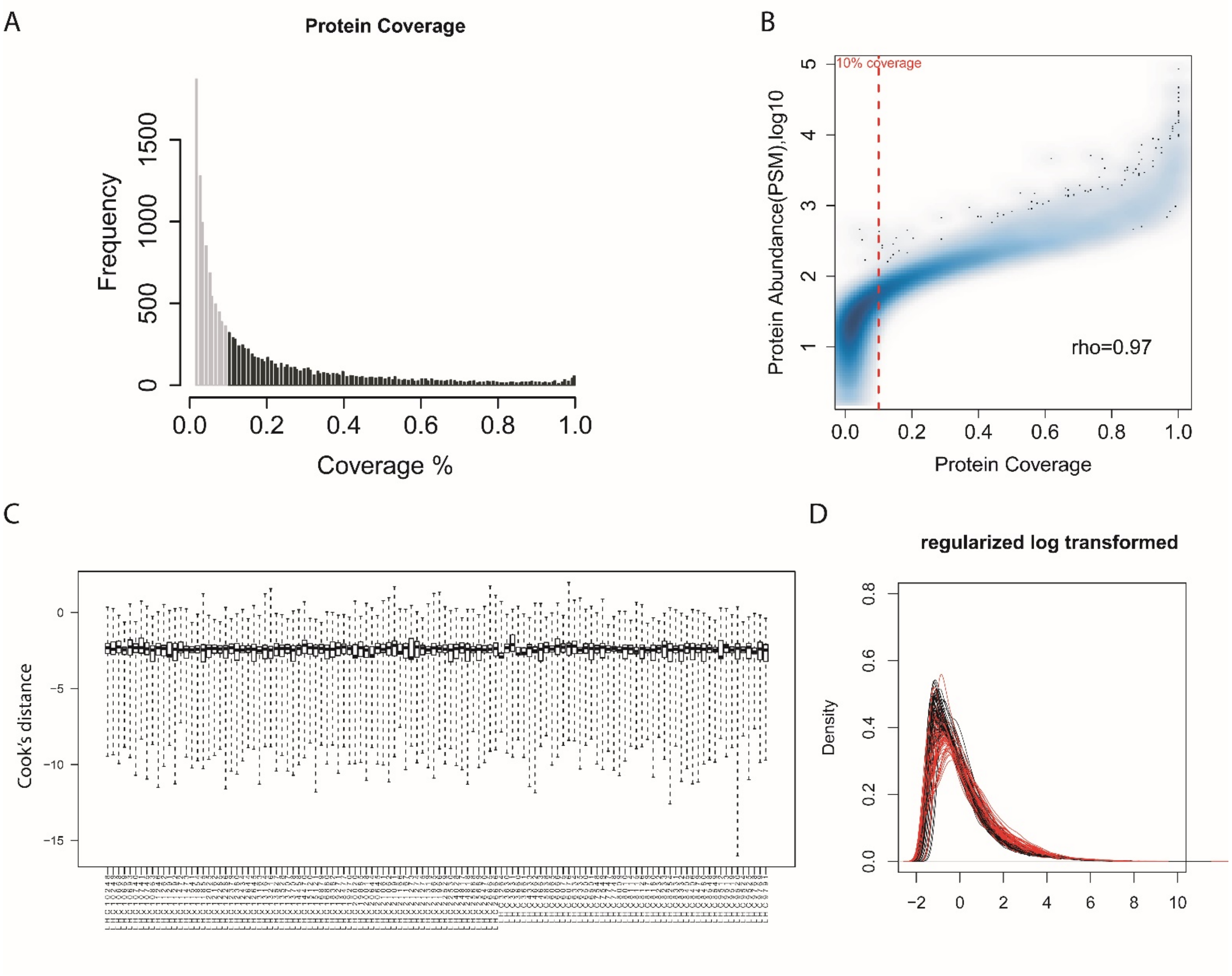

Figure S1. Processing of proteome data. **A.** Protein coverage across all samples (n = 118). Protein expressed in 12 or fewer samples (10% cut-off) were removed (grey bars). 7141 proteins (black) remained for further analysis. **B.** Correlation between protein abundance and protein coverage. A high correlation (ρ = 0.97) was observed after removing low abundance proteins. The red vertical indicates the 10% cutoff for low coverage proteins. **C.** Cook's distance across all samples. **D.** Density plot of the regularized log transformed proteomic data across all samples. (tumor samples in red, non-cancerous adjacent tissues in black).

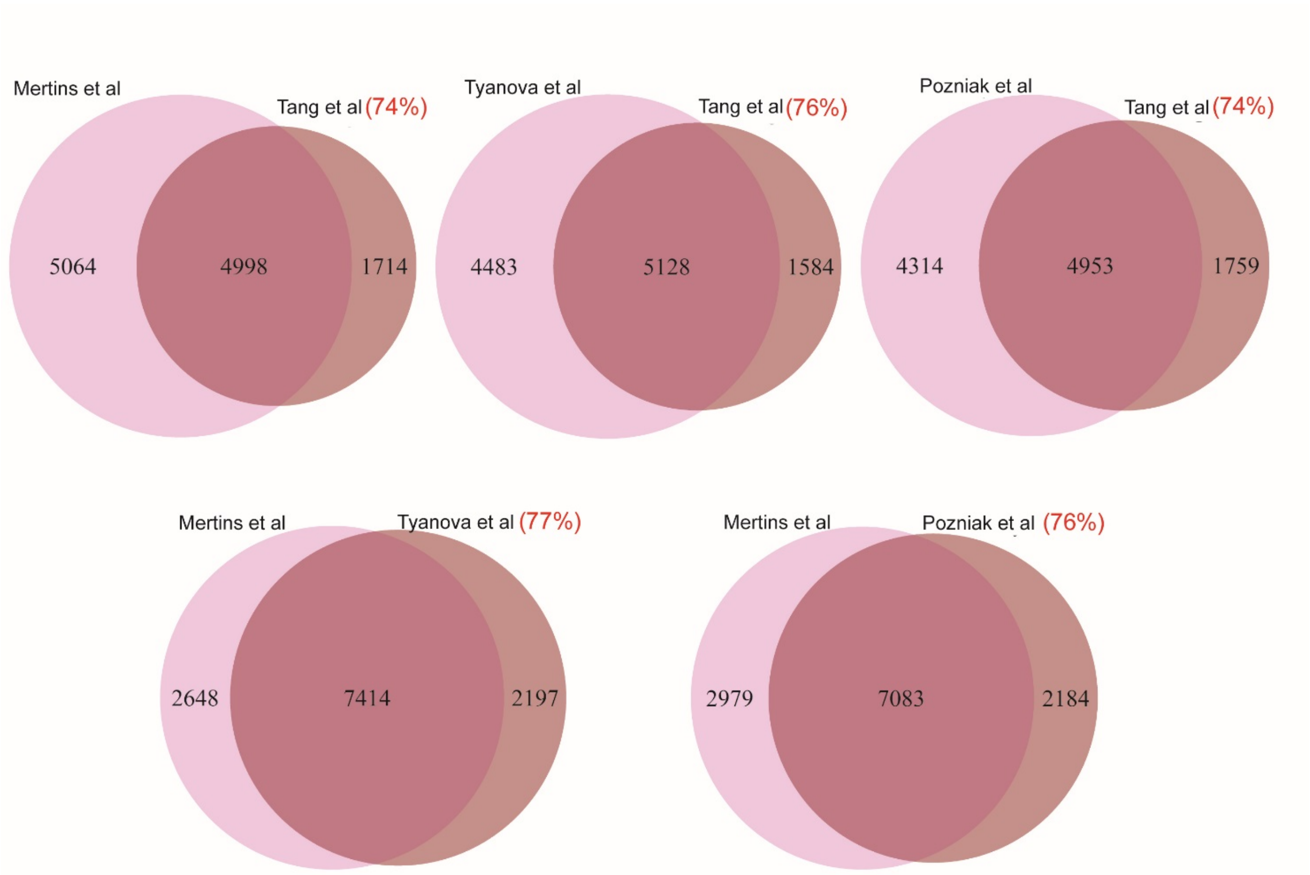

Figure S2. Overlap in identified proteins between our study (*Tang et al.*) and other proteome datasets for breast cancer. We compared our list of quantified proteins with the list of proteins reported by *Mertins et al.*, *Pozniak et al.*, and *Tyanova et al.* in their breast cancer datasets. Overall 74% to 76% of the 7141 proteins in our study were also observed in each of the other studies. This overlap is very similar to the overlap comparing *Mertins et al.* with *Tyanova et al.* or *Mertins et al.* with *Pozniak et al.* Proteome data for *Mertins et al.*, *Pozniak et al.*, and *Tyanova et al.* were either downloaded via cbioportal from the Cancer Genomics Data Server (*Mertins et al.*) or obtained from supplementray files of the publications.

Mertins P, Mani DR, Ruggles KV, Gillette MA, Clauser KR, Wang P, Wang X, Qiao JW, Cao S, Petralia F, et al: Nature 2016; ***534:****55-62.*
Pozniak Y, Balint-Lahat N, Rudolph JD, Lindskog C, Katzir R, Avivi C, Ponten F, Ruppin E, Barshack I, Geiger T: Cell Syst 2016; ***2:****172-184.*
Tyanova S, Albrechtsen R, Kronqvist P, Cox J, Mann M, Geiger T: Nat Commun 2016; ***7:****10259.*

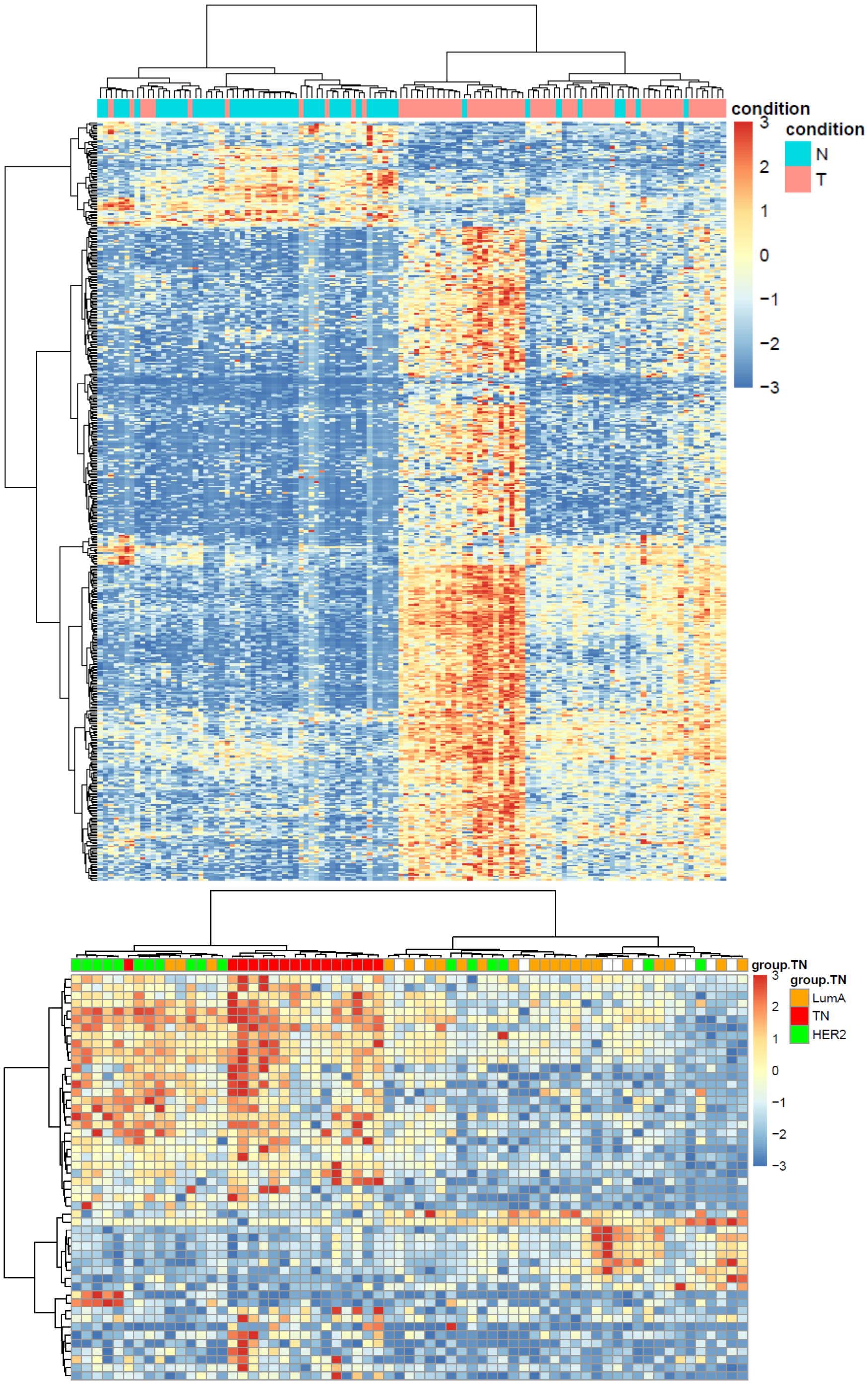

Figure S3. Proteome profiles for tumors vs. adjacent non-cancerous tissues and for tumor subtypes. **Upper panel.** Hierarchical clustering with the top 500 differentially expressed proteins between tumor and adjacent tissues. **Lower panel.** Hierarchical clustering with the top 50 differentially expressed proteins between subtypes. Each row represents a protein and each column a tissue sample. Data were Z-score scaled with blue shaded blocks representing down-regulated proteins, those in red representing up-regulated proteins. Bars above the heatmaps show the tumor (T)/normal (N) status (in upper panel) and subtype classification as luminal A (LumA), triple-negative/basal-like (TN), and Her2-positive (HER2).

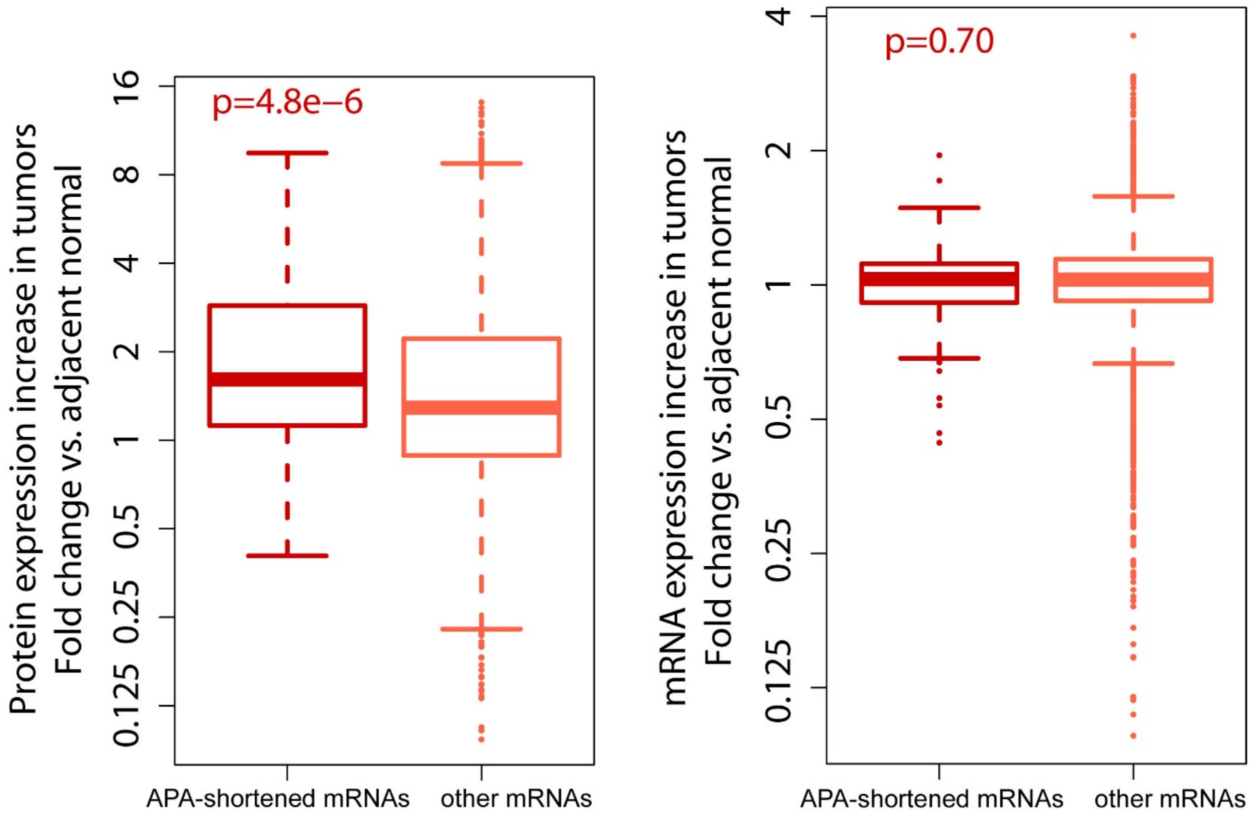

Figure S4. Increased expression of proteins in breast tumors encoded by mRNAs with shortened 3'UTR. *Xia et al.* described 382 genes with significant 3'UTR mRNA shortening in human breast tumors due to alternative polyadenylation (APA). Of these genes, we could map 193 to proteins in our study and found that these proteins have an expression increase in breast tumors (vs. adjacent non-cancerous tissue) more so than other proteins [1.77 (mean) fold increase vs. 1.41 (mean) fold increase; Wilcoxon signed rank test $P = 4.8 \times 10^{-6}$] (**left panel**), without the same increase in transcript levels (**right panel**), indicating that 3'UTR shortening leads to increased expression of proteins in breast tumors. Other mRNAs = mRNAs without alternative polyadenylation sites.

Xia Z, Donehower LA, Cooper TA, Neilson JR, Wheeler DA, Wagner EJ, Li W: Nat Commun 2014; ***5:****5274.*

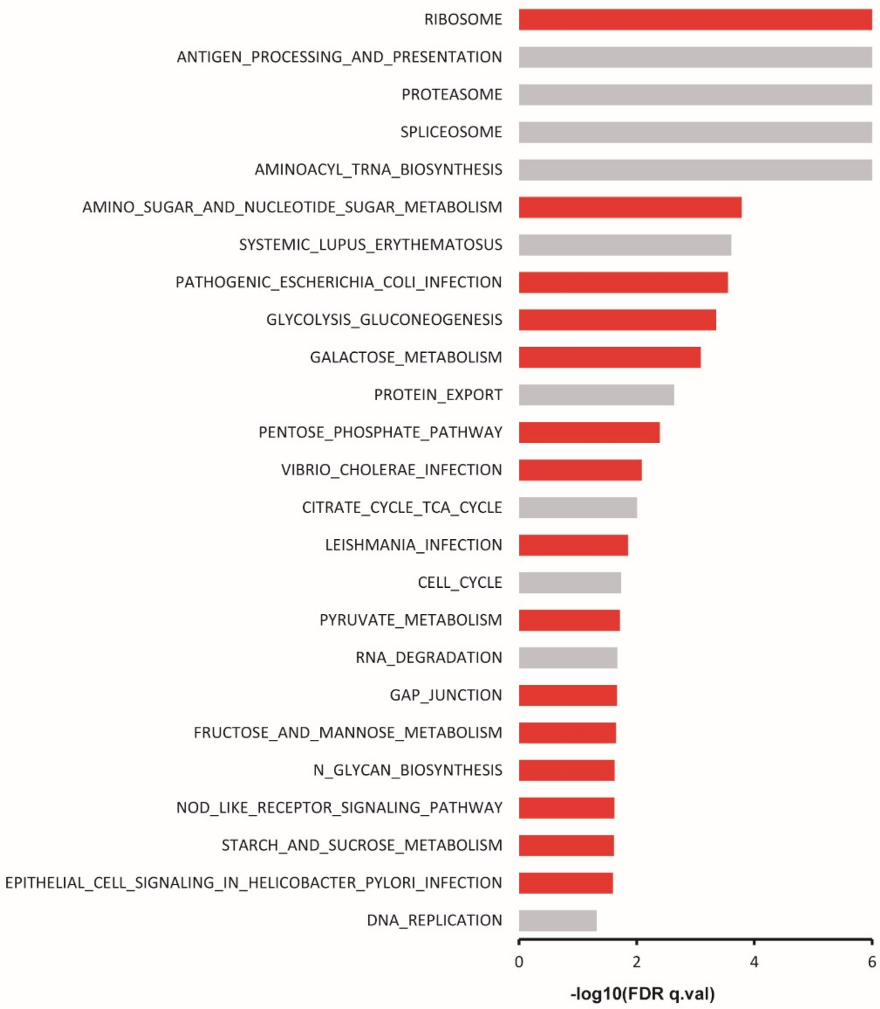

Figure S5. KEGG pathways that are significantly enriched for proteins and mRNAs that were differentially expressed between basal-like tumors and adjacent non-cancerous tissue (13 pairs). The red bars highlight KEGG pathways with enrichment for differentially expressed proteins without a similar enrichment for differentially expressed mRNAs. The grey bars indicate those pathways that were enriched for both differentially expressed proteins and mRNA in tumors. Several metabolism- and infection-related pathways were selectively enriched for differentially expressed proteins (mostly up-regulated in tumors). For the analysis, all proteins and mRNAs were ranked using Wald and t statistic and then imported into the GSEA pre-ranked module, KEGG pathway was selected as reference. KEGG pathway at a 5% FDR cutoff and |enrichment score (ES)| > 0.5 were included.

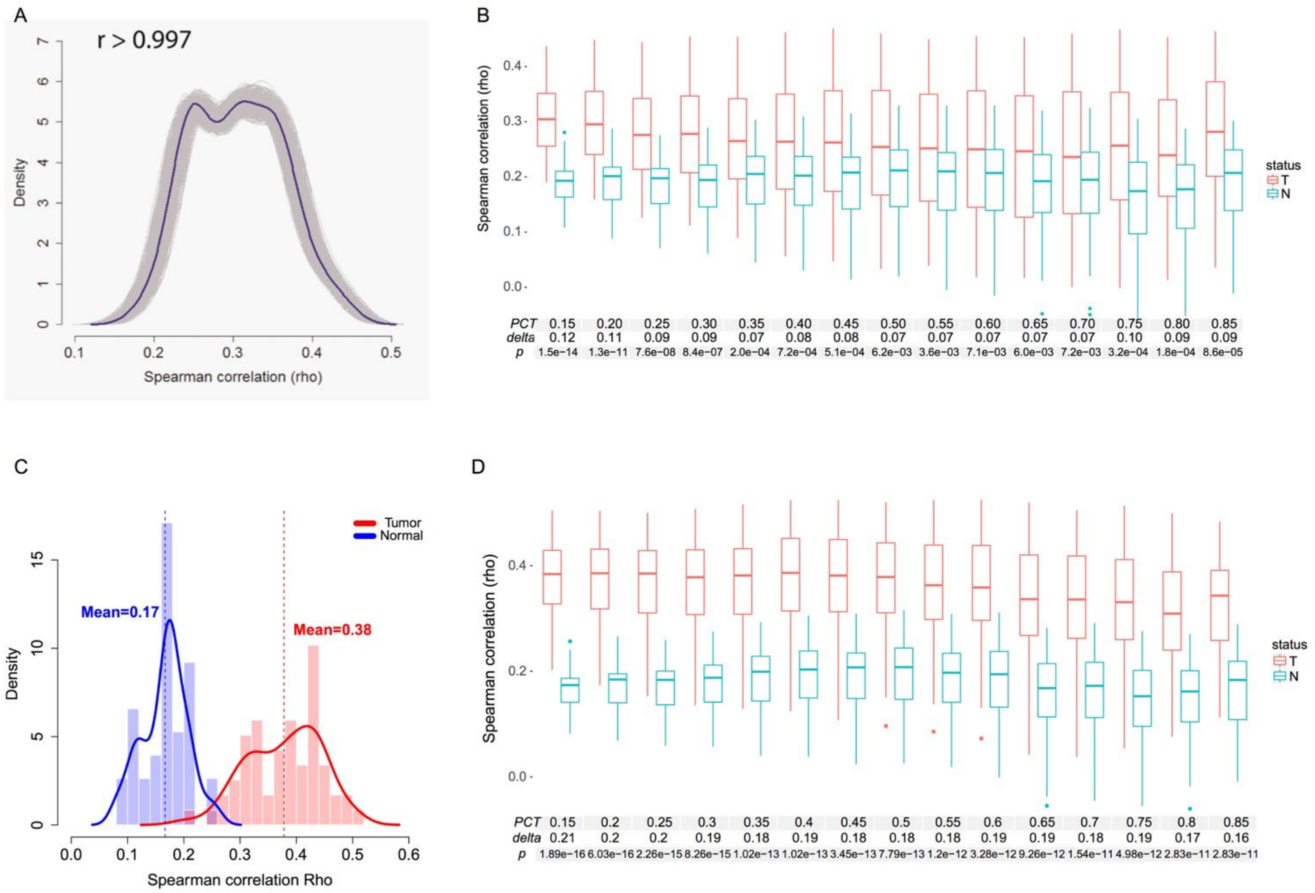

Figure S6. Robustness of the global protein-mRNA concordance estimates for breast tumors and adjacent non-cancerous tissues. A, Estimates of the global protein-mRNA concordance in breast tumors after random selection of proteome data subsets. We randomly selected 80% of the proteins from the full protein dataset and performed protein-mRNA correlation tests. The procedure was repeated 1000 times and visualized by a density plot with grey lines. Blue line shows the calculation using the full dataset. The global protein-mRNA concordance values between randomly selected subsets of data and the full dataset are very similar (r > 0.997), showing that the findings with the full dataset did not occur because of a random chance. **B**, Protein-mRNA concordance (Spearman's *rho*) differences between tumors (T) and adjacent non-cancerous tissues (N) are independent of protein abundance across samples. Shown are the concordances in relation to protein coverage with a consistently higher concordance in tumors across all coverage settings. PCT: Protein coverage from 15% to 85% in 5% increments. 15%: a protein in this group was detected in 15% or more of the tissues (tumor or adjacent non-cancerous tissues). 85%: a protein in this group was detected in at least 85% of the tissues. Delta: concordance differences between mean concordance in tumors and non-cancerous tissues. Red: tumor tissues; blue: non-cancerous tissues. Wilcoxon signed rank test. **C**, Density plot showing the global spearman correlation for protein-mRNA pairs in tumors and adjacent non-cancerous tissue. Only proteins that were expressed in both tumor and adjacent non-cancerous tissue were included in this analysis (n = 3095). Mean Spearman's correlation coefficient (*rho*) is 0.38 in tumors, which is significantly higher (P = 1.9x 10^{-16}) than the *rho* of 0.17 for adjacent non-cancerous tissues. **D**, same as **B**, but only proteins that were detectable in both tumors and adjacent non-cancerous tissues were included in this analysis.

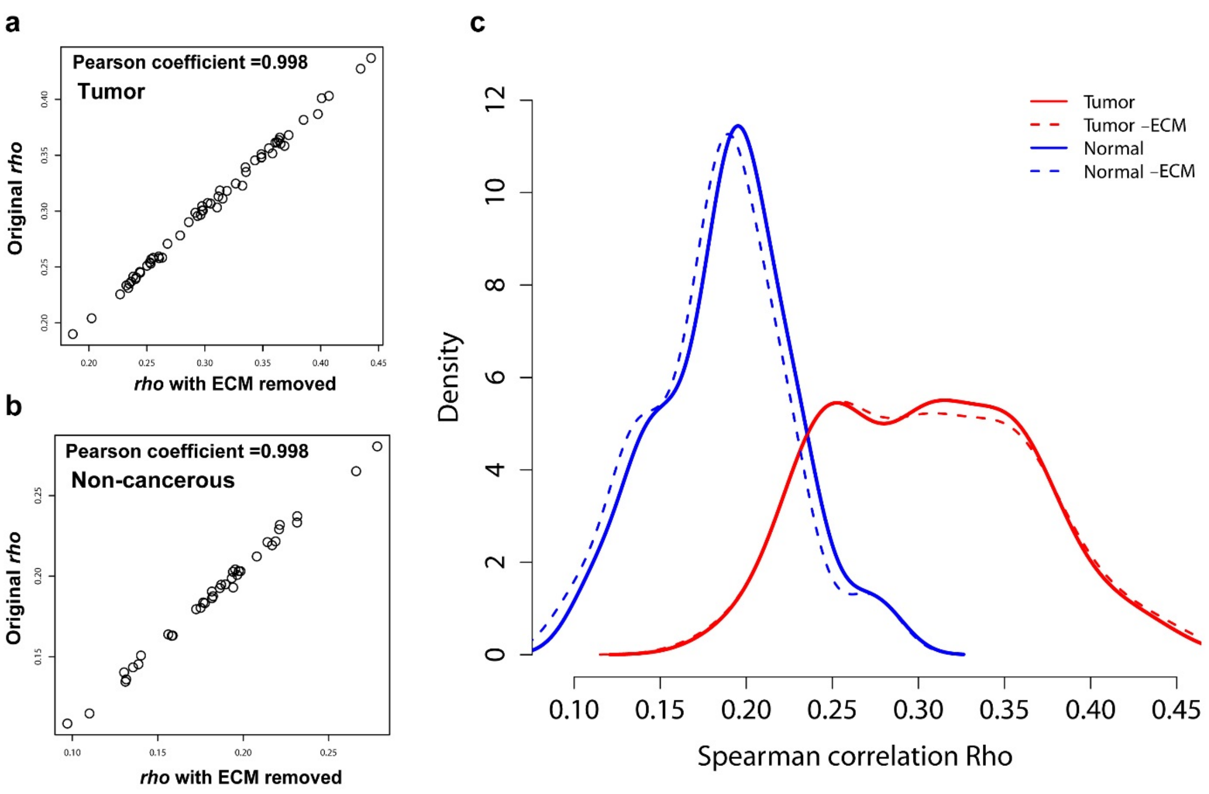

Figure S7. Exclusion of extracellular matrix proteins from the proteome dataset does not significantly alter the protein-mRNA concordance calculations for tumors or non-cancerous tissues. A, Protein-mRNA concordance calculations for all tumors with or without ('original *rho*') exclusion of 163 extracellular matrix (ECM) proteins. **B**, Protein-mRNA concordance calculations for all non-cancerous tissues with or without exclusion of 163 extracellular matrix proteins. In **A**,**B**, *rho* calculations are only slightly altered by the exclusion of these proteins. **C**, Density plot showing the global spearman correlation for protein-mRNA pairs within breast tumors and adjacent non-cancerous tissue with or without exclusion of 163 ECM proteins. Tumor-ECM/Normal-ECM shows density plots with exclusion of ECM proteins. The list of extracellular matrix proteins was downloaded from The Matrisome Project (http://matrisomeproject.mit.edu/) and included 274 genes representing collagens, ECM glycoproteins, and proteoglycans. Of those, 163 were measured in our proteome data and excluded from the analysis.

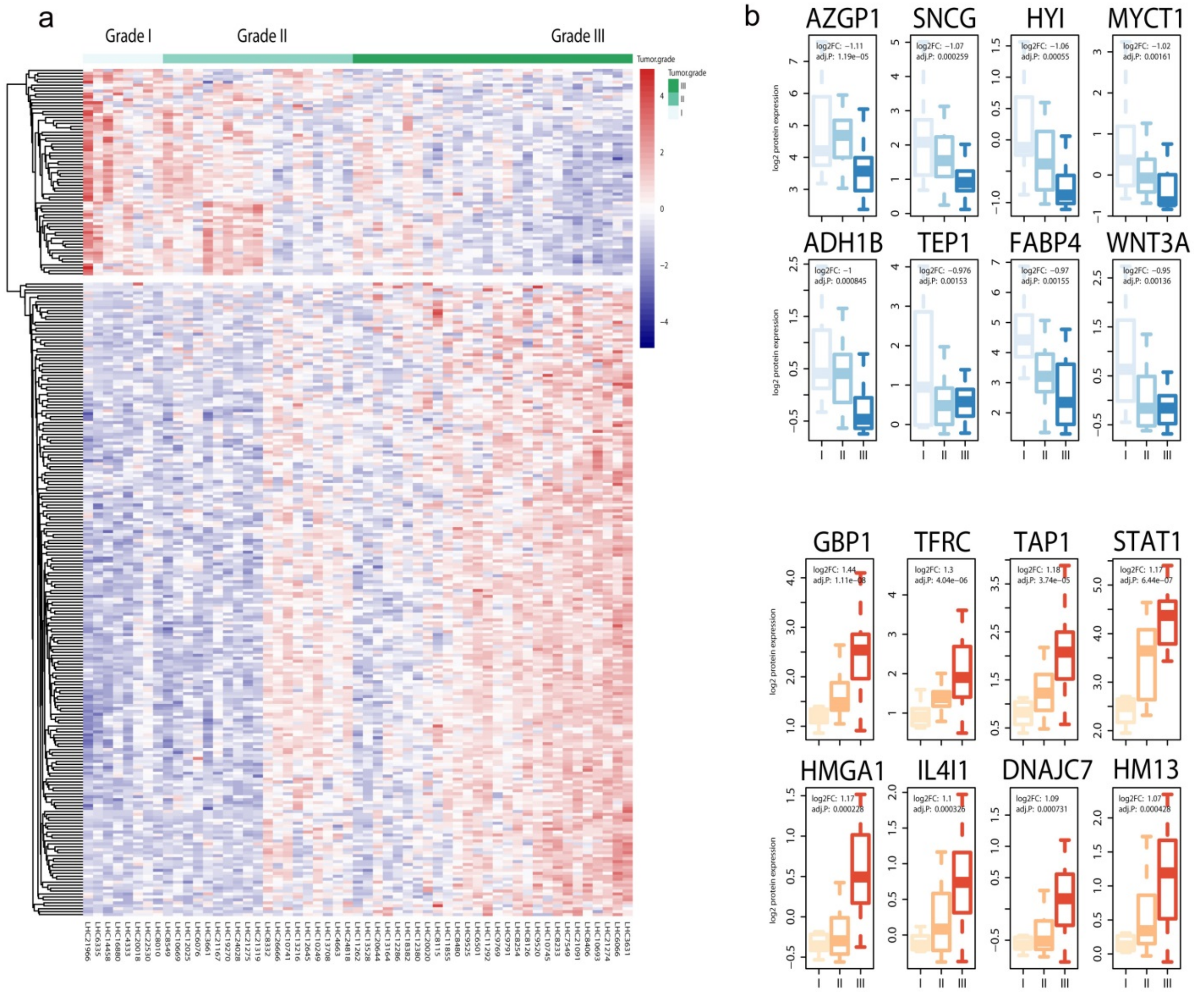

Figure S8. **Correlation between tissue protein levels and tumor grade. A**, Tissue levels of 285 proteins closely correlated with tumor grade (adj. $P_{trend} < 0.05$ for each). For visualization of the proteome data in the heatmap, we used the rlog value generated by *DESeq2*. The rlog values were row-wise scaled (centered and divided by the standard deviation), which was implemented by the R package '*pheatmap*' with scale option. **B**, Examples of proteins whose expression significantly decreased (in blue) or increased (in red) with increasing tumor grade. **Down-regulated**: AZGP1, Alpha-2-Glycoprotein; SNCG, Synuclein, Gamma (Breast Cancer-Specific Protein 1); HYI, Hydroxypyruvate Isomerase Homolog; MYCT1, Myc Target 1; ADH1B, Alcohol Dehydrogenase 1B (Class I), Beta Polypeptide; TEP1, Telomerase-Associated Protein 1; FABP4, Fatty Acid Binding Protein 4, Adipocyte; WNT3A, Wingless-Type MMTV Integration Site Family, Member 3A; **Up-regulated**: GBP1, Guanylate Binding Protein 1, Interferon-Inducible; TFRC, Transferrin Receptor; TAP1, Transporter 1, ATP-Binding Cassette, Sub-Family B (MDR/TAP); STAT1, Signal Transducer And Activator Of Transcription 1; HMGA1, High Mobility Group AT-Hook 1; IL4I1, Interleukin 4 induced 1; DNAJC7, DnaJ (Hsp40) Homolog, Subfamily C, Member 7; HM13, Histocompatibility Minor 13.

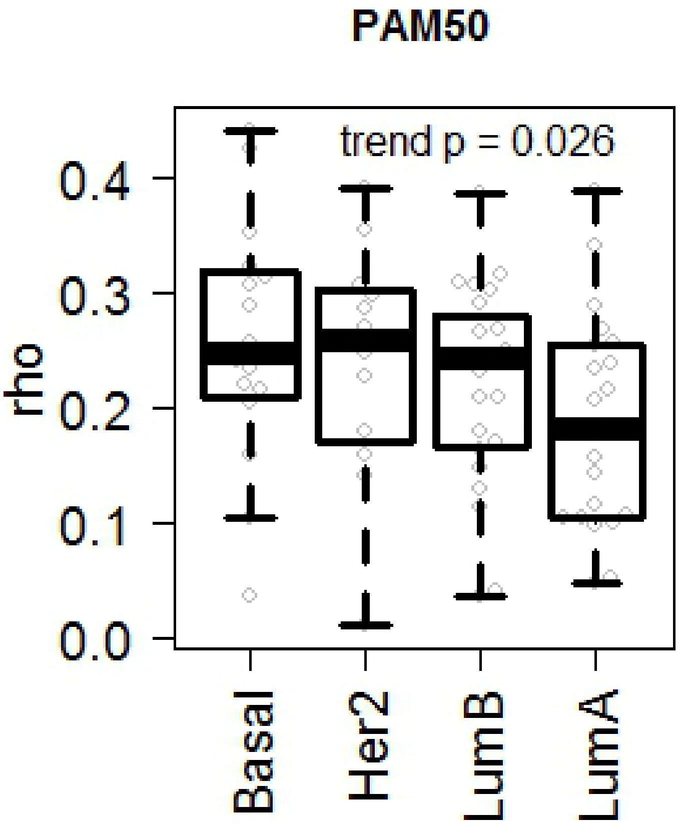

Figure S9. Correlation between steady state protein and mRNA abundance (*rho*) in breast tumors and association with PAM50-defined molecular subtypes using the CPTAC breast cancer proteomics dataset for 77 tumor samples *(Mertins et al.)*. Protein-mRNA pairs have different global correlations among breast cancer subtypes, with basal-like, HER2 and luminal B tumors having the highest correlations and luminal A tumors having the lowest correlation. Shown are box plots with minimum and maximum values (whiskers) and the median as a solid line in the box.

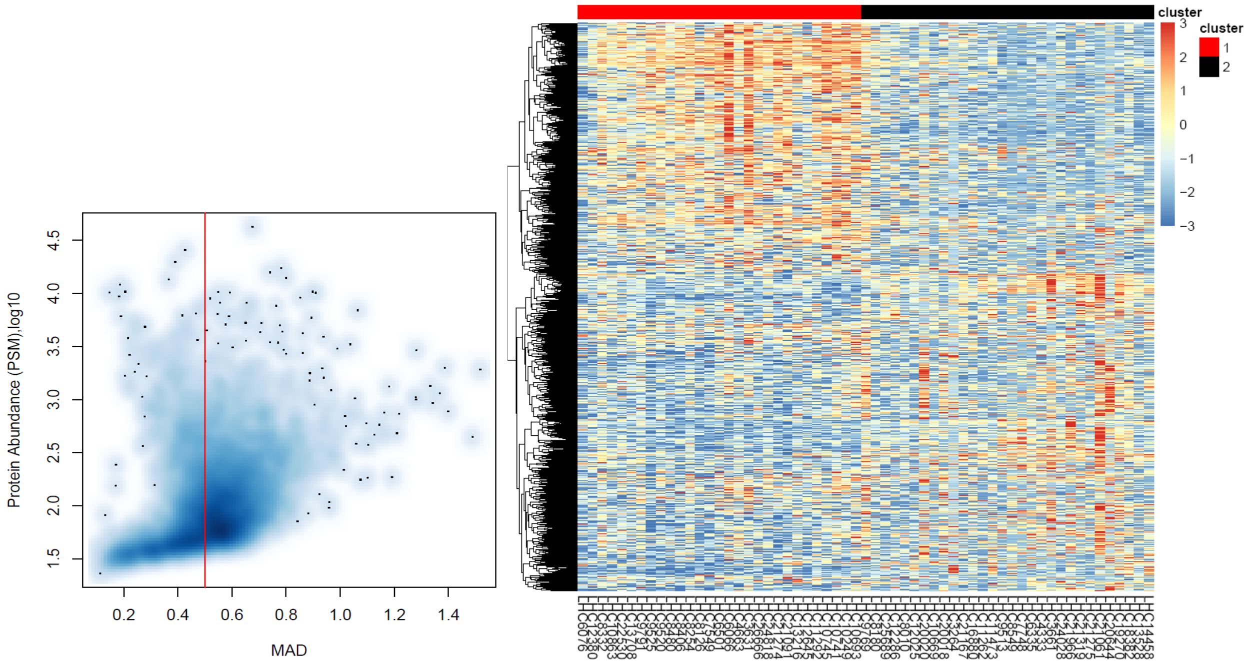

Figure S10. Non-negative Matrix Factorization (NMF) clustering of the tumor proteome data. Left panel. Scatter plot of the relationship between median absolute deviation (MAD) and protein abundance. By selecting MAD = 0.5 as the cutoff (red line), we eliminated most of the low abundance proteins. **Right panel.** Heatmap representing NMF clustering of the tumor proteome data into two groups when only high variance proteins (MAD > 0.5) were included in the analysis. We applied the NMF algorithm to all tumors and selected the 1000 proteins with the highest expression variability for clustering (see methods). In the best-fit NMF model, two distinct groups of tumors emerged. Data were Z-score scaled with blue shaded blocks representing down-regulated proteins, those in red representing up-regulated proteins.
