# Supplementary material for: Integrated proteotranscriptomics of breast cancer reveals globally increased protein-mRNA concordance associated with subtypes and survival
Source: Genome Med. 2018 Dec 3;10:94. doi: 10.1186/s13073-018-0602-x (PMC6276229; doi:10.1186/s13073-018-0602-x)
Supplement: Supplementary file 5 — Tables S3. Patient characteristics. (DOC 52 kb) [file 13073_2018_602_MOESM5_ESM.doc]

| **Table S3.** Patient characteristics | | | |  |  |
| --- | --- | --- | --- | --- | --- |
|  | | **All**  **N = 65** | **AA**^1^  **N = 31** | **EA**^2^  **N = 34** | ***P* value**^3^ |
|  | | **Mean ± SD** | | | **t test** |
| Age (years) (n=65) | | 53.9±15.2 | 54.0±15.9 | 53.9±14.8 | 0.96 |
| Tumor size (cm across) (n = 58)  West-African ancestry (%) (range among AA: 66% to 100%)  European ancestry (%) | | 4.2±2.7 | 3.8±2.4  83.4±8.7  15.8±8.9 | 4.6±3.0  2.0±4.5  97.8±4.6 | 0.30  <0.001  <0.001 |
|  | | **N** | **N** | **N** | **Fisher’s exact test** |
| ER Status | Negative | 33 | 15 | 18 |  |
|  | Triple-negative^4^ (basal-like^5^) | 16(15) | 7(7) | 9(8) |  |
|  | HER2-positive^6^ | 10 | 7 | 3 |  |
|  | other | 7 | 1 | 6 |  |
|  | Positive | 32 | 16 | 16 | 0.97^7^ |
|  | HER2-negative | 22 | 10 | 12 |  |
|  | HER2-positive (or unknown) | 7(3) | 3(3) | 4 |  |
| TNM Stage | I | 6 | 2 | 4 |  |
|  | II | 46 | 24 | 22 |  |
|  | III | 13 | 5 | 8 | 0.86 |
| Grade | 1 | 8 | 1 | 7 |  |
|  | 2 | 19 | 8 | 11 |  |
|  | 3 | 28 | 17 | 11 | 0.19^8^ |
|  | Unknown | 10 | 5 | 5 |  |

SD = standard deviation. ^1^AA=African-American, ^2^EA=European-American. Race/ethnicity by self-identification; ^3^comparing AA versus EA; ^4^Negative for estrogen, progesterone, and HER2 receptor expression; ^5^Basal-like expression signature (PAM50-defined) and/or immunohistochemistry (IHC)-based (ER-negative, HER2-negative, cytokeratin 5/6-positive or EGFR-positive); ^6^DAKO HercepTest™ IHC-positive (score 3) or IHC score 2 and HER2-enriched by gene expression; ^7^ER-negative versus ER-positive; ^8^Unknown not included
