# Supplementary material for: Integrated proteotranscriptomics of breast cancer reveals globally increased protein-mRNA concordance associated with subtypes and survival
Source: Genome Med. 2018 Dec 3;10:94. doi: 10.1186/s13073-018-0602-x (PMC6276229; doi:10.1186/s13073-018-0602-x)
Supplement: Supplementary file 8 — Tables S6. Concordance between protein-mRNA pairs in breast tumors and adjacent non-cancerous tissues within six protein abundance categories. (DOC 33 kb) [file 13073_2018_602_MOESM8_ESM.doc]

| **Table S6.** Concordance between protein-mRNA pairs in breast tumors and adjacent non-cancerous tissues within six protein abundance categories | | | | | | |
| --- | --- | --- | --- | --- | --- | --- |
|  | **Tumors** | | | | **Non-cancerous tissues** | |
| **Protein abundance*** | **Mean of *rho***** | **No. proteins** | | | **Mean of *rho*** | **No. proteins** |
| **=<50** | 0.06 | 1075 | | | 0.03 | 1797 |
| **50-100** | 0.10 | | 2093 | | 0.05 | 822 |
| **100-250** | 0.18 | | | 1490 | 0.08 | 395 |
| **250-500** | 0.26 | | | 531 | 0.14 | 148 |
| **500-1000** | 0.30 | | | 269 | 0.13 | 79 |
| **>1000** | 0.34 | | | 219 | 0.16 | 75 |

Protein abundance was categorized into six groups and the global correlation coefficient was calculated across all protein-mRNA pairs (Spearman’s *rho*) for each abundance category. * Normalized protein count within the tumor and the non-cancerous tissue groups. ** Paired Wilcoxon rank test *P*=0.031, comparing mean *rho* values for tumors with mean *rho* values for non-cancerous tissues across the six groups. Mean *rho* is significantly higher in tumors than in the adjacent non-cancerous tissue for each protein abundance category.
