# Supplementary material for: Integrated proteotranscriptomics of breast cancer reveals globally increased protein-mRNA concordance associated with subtypes and survival
Source: Genome Med. 2018 Dec 3;10:94. doi: 10.1186/s13073-018-0602-x (PMC6276229; doi:10.1186/s13073-018-0602-x)
Supplement: Supplementary file 11 — Tables S9. Association of protein-mRNA concordance with survival in the tumor proliferation score-adjusted Cox regression analysis. (DOCX 12 kb) [file 13073_2018_602_MOESM11_ESM.docx]

**Table S9**: Association of protein-mRNA concordance with survival in the tumor proliferation score-adjusted Cox regression analysis

|  | **Maryland cohort (Fig. 3H)** | | **TCGA cohort (Fig. 4C)** | | **TCGA Luminal A (Fig. 4D)** |
| --- | --- | --- | --- | --- | --- |
|  | unadjusted | Adjusted* | unadjusted | Adjusted* | Adjusted* |
| HR with 95% CI  (high vs low correlation) | 6.91 (1.50 to 31.7) | 7.59 (1.25 to 46.2) | 2.60 (1.01 to 6.65) | 2.98 (1.07 to 8.32) | 6.95 (1.28 to 38.8) |
| *P_trend_* across categories | 0.004 | 0.017 | 0.043 | 0.035 | 0.010 |

* tumor proliferation score-adjusted
